# Druggable epigenetic suppression of interferon-induced chemokine expression linked to *MYCN* amplification in neuroblastoma

**DOI:** 10.1136/jitc-2020-001335

**Published:** 2021-05-20

**Authors:** Johanna A Seier, Julia Reinhardt, Kritika Saraf, Susanna S Ng, Julian P Layer, Dillon Corvino, Kristina Althoff, Frank A Giordano, Alexander Schramm, Matthias Fischer, Michael Hölzel

**Affiliations:** 1 Institute of Experimental Oncology, Medical Faculty, University Hospital Bonn, Bonn, Germany; 2 Department of Radiation Oncology, University Hospital Bonn, Bonn, Germany; 3 Department of Medical Oncology, West German Cancer Center, University Hospital Essen, University of Duisburg-Essen, Essen, Germany; 4 Department of Experimental Pediatric Oncology, University Children's Hospital of Cologne, Faculty of Medicine, University Hospital Cologne, Cologne, Germany; 5 Center for Molecular Medicine Cologne (CMMC), Medical Faculty, University Hospital Cologne, Cologne, Germany

**Keywords:** neuroblastoma, immunomodulation, inflammation, tumor microenvironment, immunotherapy

## Abstract

**Background:**

Amplification of the *MYCN* oncogene is a molecular hallmark of aggressive neuroblastoma (NB), a childhood cancer of the sympathetic nervous system. There is evidence that *MYCN* promotes a non-inflamed and T-cell infiltration-poor (‘cold’) tumor microenvironment (TME) by suppressing interferon signaling. This may explain, at least in part, why patients with NB seem to have little benefit from single-agent immune checkpoint blockade (ICB) therapy. Targeting MYCN or its effectors could be a strategy to convert a cold TME into a ‘hot’ (inflamed) TME and improve the efficacy of ICB therapy.

**Methods:**

NB transcriptome analyses were used to identify epigenetic drivers of a T-cell infiltration-poor TME. Biological and molecular responses of NB cells to epigenetic drugs and interferon (IFN)-γ exposure were assessed by proliferation assays, immunoblotting, ELISA, qRT-PCR, RNA-seq and ChIP-qPCR as well as co-culture assays with T cells.

**Results:**

We identified H3K9 euchromatic histone-lysine methyltransferases EHMT2 and EHMT1, also known as G9a and GLP, as epigenetic effectors of the *MYCN*-driven malignant phenotype and repressors of IFN-γ transcriptional responses in NB cells. EHMT inhibitors enhanced IFN-γ-induced expression of the Th1-type chemokines *CXCL9* and *CXCL10*, key factors of T-cell recruitment into the TME. In *MYCN*-amplified NB cells, co-inhibition of EZH2 (enhancer of zeste homologue 2), a H3K27 histone methyltransferase cooperating with EHMTs, was needed for strong transcriptional responses to IFN-γ, in line with histone mark changes at *CXCL9* and *CXCL10* chemokine gene loci. EHMT and EZH2 inhibitor response gene signatures from NB cells were established as surrogate measures and revealed high EHMT and EZH2 activity in *MYCN*-amplified high-risk NBs with a cold immune phenotype.

**Conclusion:**

Our results delineate a strategy for targeted epigenetic immunomodulation of high-risk NBs, whereby EHMT inhibitors alone or in combination with EZH2 inhibitors (in particular, *MYCN*-amplified NBs) could promote a T-cell-infiltrated TME via enhanced Th1-type chemokine expression.

## Background

Neuroblastoma (NB) is an extracranial solid tumor of early childhood that originates from the developing sympathetic nervous system. Activation of telomere maintenance mechanisms characterizes high-risk NB,^1^ and additional alterations in the p53 or RAS pathway, known to play a role in NB pathogenesis,^2–5^ further worsen disease outcome. Amplification of the *MYCN* gene, which encodes for the oncogenic transcription factor MYCN (also known as N-Myc), was the first genomic aberration found to be associated with poor prognosis.^6^ Despite these molecular insights, high-risk NB remains a major challenge in pediatric oncology. Serious long-term side effects caused by intensive radiochemotherapy emphasize the need for novel treatment approaches with reduced toxicity.^7 8^

Monoclonal antibodies directed against programmed cell death 1 (PD-1), programmed cell death 1 ligand 1 (PD-L1) or cytotoxic T-lymphocyte associated protein 4 (CTLA-4), also known as immune checkpoint inhibitors, have significantly improved the therapy of various cancers. Immune checkpoint inhibitors reinvigorate anti-tumor immunity depending on various parameters including tumor immunogenicity and the presence of tumor-infiltrating T cells.^9^ The latter is also known as a T-cell-inflamed or ‘hot’ tumor microenvironment (TME). A hot TME is further characterized by high interferon (IFN) pathway activity,^10^ a signaling cascade that plays a central role in the coordination of immune responses.^11^ In contrast, a T-cell infiltration-poor, ‘cold’ TME is usually associated with poor responses to immune checkpoint blockade (ICB) therapy. Recently, we and others showed that *MYCN*-amplified NBs are characterized by a ‘cold’ TME. Mechanistically, MYCN suppresses IFN activity and chemokine expression in NB cells, thus providing a possible explanation for this association,^12–16^ in line with the emerging notion that ICB therapy seems to be ineffective in patients with high-risk NB, although clinical data remain limited.^17 18^

We previously proposed that STING agonists could be promising drugs to induce conversion of ‘cold’ to ‘hot’ TMEs in NB.^12^ The cGAS/STING pathway plays a central role in the sensing of cytosolic DNA and its activation triggers a strong type I IFN response.^19^ Recently, Wang-Bishop *et al*^20^ demonstrated that intratumoral application of STING-activating nanoparticles induced immunogenic cell death, chemokine release and a T-cell-inflamed ‘hot’ TME in transplantable mouse models of NB improving ICB therapy. Depending on the clinical context, however, intratumoral injections of STING agonists could be challenging. Advanced strategies of tumor-targeted drug delivery could overcome this issue,^21^ but also alternative treatment strategies are needed.

Here, we tested the concept that epigenetic modifiers could be attractive drug targets to rewire NB immune phenotypes. By exploring NB transcriptome data, we found that euchromatic histone lysine methyltransferases *EHMT2* and *EHMT1* (also known as *G9a* and *GLP*) were negatively correlated with the expression of Th1-type chemokines (*CXCL9*, *CXCL10*), key drivers of a T-cell-inflamed ‘hot’ TME. *EHMT* expression was higher in *MYCN*-amplified human NBs and pharmacological inhibition of EHMTs enhanced IFN-γ-induced chemokine expression. In *MYCN*-amplified NB cells, robust IFN-γ responses required co-inhibition of EZH2 (enhancer of zeste homologue 2), a histone methyltransferase cooperating with EHMTs in gene repression. Finally, gene signatures from inhibitor-treated NB cells served as surrogate measures of high EHMT and EZH2 activity in patient samples from high-risk NBs. These gene signatures showed a strong association with *MYCN* amplification and a T-cell-poor, cold TME delineating tailored strategies for epigenetic immunomodulation of high-risk NBs.

## Methods

### Cell culture

All commonly used human NB cell lines (SK-N-BE, IMR-32, NMB, CHP-134, GI-M-EN, SK-N-AS, SK-N-FI, SH-SY5Y) were available in our laboratory.^2^ The cell line mNB-A1 was derived from the transgenic NB mouse model LSL-MYCN;Dbh-iCre.^22^ The NHO2A cell line was derived from the transgenic TH-MYCN NB model.^23^ None of the used cell lines are listed in the ICLAC database of misidentified cancer cell lines. All cell lines were cultured in a humidified incubator with 5% CO_2_ at 37 °C. Cell lines were cultured in RPMI-1640 medium with 10% fetal bovine serum, 2 mM glutamine, 100 U/mL penicillin and 100 mg/mL streptomycin (all Gibco, Thermo Fisher Scientific, Waltham, Massachusetts, USA). SK-N-FI was cultured in DMEM/F12 medium (Gibco, Thermo Fisher Scientific). mNB-A1 was cultured with the addition of B-27 and N-2 supplements (all Gibco, Thermo Fisher Scientific). All cell lines were negative for mycoplasma contamination. Testing for mycoplasma was performed by PCR on a monthly basis.

### Epigenetic inhibitor treatment in combination with interferon responses

All inhibitors were dissolved in DMSO, which was used as vehicle control in all assays. Human and mouse NB cells were seeded in 12-well plates the day before treatment to adhere at 60%–70% confluency. Either EHMT inhibitor UNC-0638 (Cayman Chemical, #10734) or BIX-01294 (AdipoGen, #AG-CR1-0051) were added to the culture media at concentrations of 1 µM–5 µM. For titration experiments, recombinant human IFN-γ (PeproTech, #300-02) was added directly to the culture media 72 hours after inhibitor exposure, and cells were incubated for an additional 24 hours. Cells were lysed for total RNA or protein isolation. Supernatants were recovered for ELISA. EZH1/2 and PRC2 inhibitors: Human NB cells were seeded in 12-well plates the day before treatment to adhere at 70%–80% confluency. The four different small molecule inhibitors CPI-1205 (Selleck Chemicals, #S8353), EED226 (Selleck Chemicals, #S8496), GSK503 (Cayman Chemical, #18531) and EPZ011989 (MedChemExpress, #HY-16986) were added to the culture media at a concentration of 3 µM for 96 hours. Cells were lysed for protein isolation. EHMT/EZH2 inhibitors and interferon responses: human NB cell lines SK-N-BE and IMR-32 were seeded in 12-well plates the day before treatment to adhere at 60%–70% confluency. Either 2 µM of EHMT inhibitor UNC-0638 (Cayman Chemical, #10734), 3 µM of EZH2 inhibitor EPZ011989 (MedChemExpress, #HY-16986) or the combination of both (UNC-0638 2 µM plus EPZ011989 3 µM) were added to culture media for 7 days with media changes every 3 days. At day 6, recombinant human IFN-γ (PeproTech, #300-02) was added and cells were incubated for 24 hours before lysis to obtain cells total RNA or protein. Culture media supernatants were recovered for ELISA.

### Cell proliferation assays

Cells were seeded in 12-well plates at low density, treated with inhibitors as indicated and fixed once the control well reached confluency, using 4% formaldehyde. Plates were washed with distilled H_2_O and stained for 30 min using 0.05% crystal violet. Stained plates were scanned at 800 nm using the Odyssey Sa Imaging System (LI-COR Biosciences, Lincoln, Nebraska, USA) and signal intensities were used as surrogate measures for the quantification of relative cell numbers.

### Immunoblot analysis

Cells were lysed in 1x Laemmli buffer (2000 cells per μL) and incubated for 5 min at 95 °C. Cell lysates were separated by 8%, 10% or 15% SDS-PAGE and transferred to a nitrocellulose membrane (GE Healthcare) by wet blotting for 90 min at 70 V (both systems by Bio-Rad). Membranes were blocked with 5% bovine serum albumin (BSA) (GE Healthcare) in Tris-buffered saline (TBS) with 0.5% Tween for 1 hour on a shaker and then probed with primary antibodies diluted in 5% BSA in TBS with 0.5% Tween at 4 °C overnight. On the following day, membranes were probed with IRDye680LT and IRDye800CW secondary antibodies diluted in 3% BSA in TBS with 0.5% Tween. Proteins were detected by measuring at 700 nm and 800 nm wavelengths using the Odyssey Sa Imaging System (LI-COR Biosciences). Antibodies and markers used were as follows: β-actin (#47778, Santa Cruz Biotechnology), N-Myc (#53993, Santa Cruz Biotechnology), c-Myc (#ab168727, Abcam), EZH1 (#42088, CST), EZH2(#5246, CST), EHMT1 (#35005, CST), G9a/EHMT2 (#68851S, CST), H3K9me2 (#9753, CST), H3K27me3 (#9733, CST), Broad Range Markers (#2361, Santa Cruz Biotechnology). Western blot quantification was performed by measuring protein band density using the Image Studio Lite program, V.5.21 (LI-COR Biosciences). Optical density measurement was performed with standardized background correction for each gel and values were normalized against internal β-actin expression of each blot. Data was processed to show fold change of protein expression compared with positive control.

### Enzyme-linked immunosorbent assay

Supernatants of stimulated cell cultures were collected and centrifuged at 300g for 3 min to clear of debris or cells. Samples were stored at −80 °C and re-thawed on ice prior to analysis. ELISA kits for human CXCL10 (R&D DuoSet, #DY266-05) and for murine CXCL10 (R&D DuoSet, #DY466-05) were used according to manufacturer’s instructions. The absorbance of the samples was measured using Infinite M200 PRO (Tecan). For wavelength correction, absorbance measured at 570 nm was subtracted from the absorbance measured at 450 nm. The concentration of chemokines in the supernatants was calculated based on the standard curve generated using the provided chemokine standard.

### PBMC isolation

Peripheral blood mononuclear cells (PBMCs) were isolated by diluting whole blood with an equal part of RPMI 1640 (Gibco, Thermo Fisher Scientific). Diluted blood was then overlaid onto Ficoll-Paque PLUS (GE Healthcare, Chicago, Illinois, USA) and centrifuged at 450g for 30 min at room temperature. PBMCs were harvested from the interphase using a transfer pipette and added to RPMI 1640. PBMCs were washed twice in RPMI 1640 by centrifuging at 400g for 10 min and discarding the supernatant, following which cell pellets were resuspended in RPMI 1640 containing 10% fetal calf serum (FCS; Gibco, Thermo Fisher Scientific) and enumerated. PBMCs were frozen in freezing media (RPMI 1640+10% DMSO (Carl Roth, Karlsruhe)).

### T-cell activation

Ninety-six-well U-bottom plates were coated with 10 µg/mL Ultra-LEAF anti-human CD3 antibody (clone: OKT3; BioLegend, San Diego, California, USA) diluted in Dulbecco's phosphate-buffered saline (DPBS) (Gibco, Thermo Fisher Scientific) by incubating at 37°C for 2 hours. PBMCs were thawed in a water bath at 37°C, washed once in RPMI 1640 and resuspended in human T-cell media (RPMI 1640 containing 10% FCS, penicillin and streptomycin, GlutaMAX, non-essential amino acids, 0.05 mM 2-mercaptoethanol, 1 mM sodium pyruvate (all from Gibco, Thermo Fisher Scientific) and 5 mM HEPES (Carl Roth)). PBMCs were left to rest at 37°C for 2 hours and enumerated on the Countess II FL (Thermo Fisher Scientific). T cells were isolated using the Pan-T cell Isolation Kit, human (Miltenyi Biotec, Bergisch Gladbach), according to manufacturer’s instructions and activated in plates previously coated with anti-human CD3 as described above in the presence of 5 µg/mL anti-human CD28 antibody (clone: CD28.2; BioLegend) and 100 U/mL Proleukin (Novartis Pharma, Basel, Switzerland) for 24 hours at 37°C. Non-activated T cells were incubated for the same duration in human T-cell media. Validation of T-cell activation was performed by flow cytometry assessment of CD69, CXCR3 and IFN-γ expression. Briefly, BD GolgiPlug Protein Transport Inhibitor (BD Biosciences, San Jose, California, USA) was added to T cells according to manufacturer’s instructions after 20 hours of activation. Four hours later, T cells were stained with Human TruStain FcX, Zombie Aqua, Pacific Blue anti-human TCR α/β (IP26), FITC anti-human CD19 (4G7), FITC anti-human CD56 (NCAM; HCD56), FITC anti-human CD14 (63D3), Spark Blue 550 anti-human CD8 (SK1), PE/Dazzle 594 anti-human CD183 (CXCR3; G025H7), APC anti-human CD69 (FN50), Alexa Fluor 700 anti-human CD45 (2D1) and APC/Cy7 anti-human CD4 (SK3) (all from BioLegend). Cells were subsequently fixed and permeabilized using the BD Fixation/Permeabilization Solution Kit according to manufacturer’s instructions and stained with PE/Cy7 anti-human IFN-γ (4S.B3). Samples were acquired on the Cytek Aurora 3 L (Cytek Biosciences, Fremont, California, USA) and analyzed on FlowJo (BD Biosciences).

### T-cell co-culture assays with SK-N-BE or SH-SY5Y cells

SK-N-BE or SH-SY5Y cells were treated with either DMSO or UNC+EPZ as described above. On day 6 of treatment, non-activated or activated T cells were washed once, resuspended in RPMI 1640+10% FCS, then co-cultured with either cell line at a ratio of NB cells:T cells of 10:1. In the case of activated T cells, the ratio of NB cells:CD69^+^IFN-γ^+^ T cells was approximately 100:1. Plates were centrifuged briefly at 100g and incubated for 24 hours at 37°C. Supernatants were collected for analysis of CXCL10 levels by ELISA as described above. Post-co-culture, SK-N-BE and SH-SY5Y cells were analyzed by RT-qPCR for *CXCL9* and *CXCL10* expression or flow cytometry for HLA-A, B, C. Flow cytometry analysis of SK-N-BE and SH-SY5Y cells involved surface staining for Human TruStain FcX, Zombie Aqua, phycoerythrin (PE) anti-human HLA-A, B, C (W6/32) and Alexa Fluor 700 anti-human CD45 (2D1), fixation and permeabilization using the eBioscience Foxp3/Transcription Factor Staining Buffer Set according to manufacturer’s instructions. Samples were acquired on the Cytek Aurora 3L, analyzed on FlowJo (V.10.7.1, Mac OS X) and graphed on Prism 9 (GraphPad, San Diego, California, USA).

### RNA isolation, cDNA synthesis and quantitative real-time PCR (qRT-PCR)

Treated cells were lysed with RLT buffer (Qiagen) and RNA was isolated with Zymo Spin II columns (Zymo Research). cDNA synthesis was performed with the All-in-One cDNA Synthesis SuperMix (Biotools). qRT-PCR reactions from at least biological triplicates were always prepared in technical duplicates in a total volume of 10 µL containing EvaGreen (BioBudget), primers and RNase-free H_2_O. qRT-PCR was performed using a Roche LC480 according to the manufacturer’s instructions. Human and mouse samples were quantified by normalization to the housekeeping genes *UBC* and *Ubc*, respectively. Sequences of qRT-PCR primer pairs (Microsynth, Switzerland) are as follows: human *CXCL10*: forward GCAGGTACAGCGTACGGTTC, reverse CAGCAGAGGAACCTCCAGTC; human *UBC*: forward CCATCACACCCAAGAACAAGCACA, reverse AGGCAAGACCATCACCTTGGACG; mouse *Cxcl10*: forward CCTATGGCCCTCATTCTCAC, reverse CTCATCCTGCTGGGTCTGAG; mouse *Ubc*: forward CCATCACACCCAAGAACAAGCACA, reverse AGGCAAGACCATCACCTTGGACG.

### Chromatin immunoprecipitation qPCR

Chromatin immunoprecipitation (ChIP) assays were performed using a SimpleChIP Enzymatic Chromatin IP kit (#9003, Cell Signaling Technologies (CST)). SK-N-BE cells were treated with 2 µM UNC-0638, 3 µM EPZ011989, the combination of both inhibitors or DMSO as untreated control. Media and inhibitor were changed every 2 days. After 6 days, cells were cross-linked with 37% formaldehyde at a final concentration of 1% at room temperature for 10 min. Fragmented chromatin was treated with micrococcal nuclease and subjected to sonication. ChIP was performed with rabbit anti-histone H3 as a technical positive control (#4620, CST), rabbit Di-Methyl-Histone H3 (Lys9) antibody (#4658, CST), rabbit Tri-Methyl-Histone H3 (Lys27) antibody (#9733, CST) and normal rabbit IgG as a negative control (#2729, CST). After reverse cross-linking and DNA purification, immunoprecipitated DNA was quantified by real-time PCR with primers for *CXCL9*, *CXCL10* and *SDAD1: CXCL9* #1 forward CCTACACAATTCACTGAACCTCCC, reverse CCATGAAGAAAGGGAACGGTGAAG; *CXCL9* #2 forward CATTCTCCTGGCAACCTTGCTTAC, reverse AAAGTCACCCTCTCCAGAACTCAG; *CXCL9* #3 forward GGAATGGAACTGGTGTTGGTGTTG, reverse GGAGGAGAGAGGATGAGGATGAAAG; *CXCL9* #4 forward GGCAGAACATTCACCTTTATCTGGC, reverse TCCTCTTGGGCATCATCTTGCTG; *CXCL10* #1 forward GCTGTACTTCAAGGTTGACTGGT, reverse AAGTATGTTACCACCACGCCCTC; *CXCL10* #2 forward GAGGGCGTGGTGGTAACATACTT, reverse CTGAAAGCAGTTAGCAAGGAAAGG; *CXCL10* #3 forward GTTTGATTCATGGTGCTGAGACTG, reverse CAGAATTAGGGAGGGAAAATGGC; *CXCL10* #4 forward CGTGGGGCTAGTGTGCCATATTT, reverse TCGAGTCTGCAACATGGGACTTC; *SDAD1* #1 forward GCTAACACTATTTACCCGCCTTGAG, reverse TGAGGATTACAGTGAGGAGGATGAG; *SDAD1* #2 forward CAGCATGGATGAGTCAGTAGGCAT, reverse AGGAGGAATAGTGGGTCTTGTGG; *SDAD1* #3 forward GAGGGAAGTGAGAAGGGCAATGA, reverse ACAGGTGCTTCCTTCAGACACTC; *SDAD1* #4 forward GGTCAGTACACAAAGCCCATCAC, reverse CCAGGATTCAAACTTACCGGAGAC. Using *RPL30* exon 3 (#7014, CST) as positive control. Quantification was based on percentage of input.

### 3′mRNA-sequencing and initial processing

NB cells were lysed in RLT buffer (Qiagen) and total RNA was isolated using Zymo I spin columns (Zymo Research) followed by elution in RNAase-free H_2_O. 3′mRNA-seq library preparation was performed by the University Hospital Bonn (UKB) next generation sequencing (NGS) core facility using the forward QuantSeq 3′mRNA-seq Library Prep Kit for Illumina (Lexogen GmbH, Austria) according to the manufacturer’s protocol. Size distribution and library yield after the PCR step were determined using the D1000 high sensitivity tapestation (Agilent) prior to pooling of the barcoded libraries. The pooled libraries were loaded onto the Illumina HiSeq2500 platform and analyzed by a 50-cycle high-output run. Computational analysis was performed using the R-based Bioconductor computing environment. FASTQ files were aligned to the Hg38 human reference genome using the Rsubread aligner package.^24^ To adjust the alignment procedure to 3′mRNA-seq data, the Rsubread align function was executed without trimming but allowing for mismatches in the initial cycles. Only reads with at least 45 bases in length were included in the analysis. Initial mapping using the Rsubread algorithm (‘align’) allowed for ambiguous mapping (maximum two genomic sites to allow for junction reads), but gene level summary with the ‘featureCounts’ methods was set to unique mapping. The ‘voom’ method of the limma package was used for normalization and linear modeling.^25^ The mRNA expression values were transformed to log2 values of read counts per million (log2 cpm).

### Gene signature and differential gene expression analyses

Gene set enrichment analysis (GSEA) was performed using a Java-based stand-alone version. Gene set collections were obtained from the Molecular Signature Database (MSigDb v.7.2, https://www.gsea-msigdb.org). The preranked gene list mode was used for the analyses with 1000 permutations and default settings. GSEA plots were generated with the R-function replotGSEA accessible via https://github.com/PeeperLab/Rtoolbox/blob/master/R/ReplotGSEA.R. Differential gene expression analysis for drug responses was performed using the limma functions ‘lmFit’, ‘eBayes’ (eBayes moderated t-test statistics) and ‘topTable’.^26^ The contrast design was drug versus vehicle. Raw p values were corrected for multiple testing using the Benjamini and Hochberg method (false discovery rate, FDR). Final drug response signatures (Up_by_Drug, Down_by_Drug; Drug: UNC-0638, EPZ011989 or UNC-0638+EPZ011989) were obtained by taking the overlap of differentially upregulated or downregulated genes with FDR<0.05 from each cell line (SK-N-BE, IMR-32). Raw sequencing data is available through the European Nucleotide Archive under the accession numbers PRJEB38901 and PRJEB42679.

### Transcriptome analysis of published NB patient cohort (NB498 cohort)

Preprocessed, normalized and log2-transformed RNA-seq data (GEO accession numbers: GSE49711/GSE62564) were accessed through the R2 Genomics Platform (http://r2.amc.nl). Clinical annotation and *MYCN* status were obtained from the Gene Expression Omnibus (GEO) via the respective accession numbers. Data were imported into the R/Bioconductor computing platform. To calculate transcriptome-based surrogate EHMT, EZH2 or EHMT/EZH2 activity scores (ratioed signatures) for individual NB patient samples, we divided the average (mean) of log2 expression values of ‘Down_by_Drug’ genes by the average (mean) of log2 expression values of ‘Up_by_Drug’ genes for each of the samples. ‘Drug’ represents UNC-0638, EPZ011989 or UNC-0638+EPZ011989. NB patient samples were ranked by increasing activity scores. The use of ratio-based signatures (scores) for tumor transcriptomes datasets was described previously.^27^

### Analysis of public ChIP-seq data sets of NB cell lines

MYCN, MYC and histone ChIP-seq data (GSE138295 and GSE138314) were download as bigwig (bw) files from GEO archive.^28^ After bed file conversion, data sets were imported into the R/Bioconductor computing environment. Analysis and visualization of data were performed as described within: https://www.bioconductor.org/help/course-materials/2016/CSAMA/lab-5-chipseq/Epigenetics.html. Essential packages for the analysis were ‘chipseq’, ‘Gviz’, ‘GenomicRanges’, ‘rtracklayer’ and ‘IRanges’. Hg19 genome build was used as reference.

### Gene expression analysis of CCLE

*EHMT2* and *EHMT1* gene expression values (RNA-seq) and sample annotation of Cancer Cell Line Encyclopedia (CCLE) were accessed via the public link www.broadinstitute.org/ccle.^29^ RNA-seq expression values were on log2 scale.

### Statistical tests

Statistical tests were performed using GraphPad Prism 8 or R (V.3.3.2, x86_64-pc-linux-gnu) and specified in the figure legends. Assuming a normal distribution, two-way Student’s t-test (parametric) was used to determine statistical significance and differences, otherwise non-parametric tests were used. Corrections for multiple testing were performed when applicable (Benjamini and Hochberg method). References to p values are specified for each figure in the corresponding figure legends.

## Results

**Figure 1:**
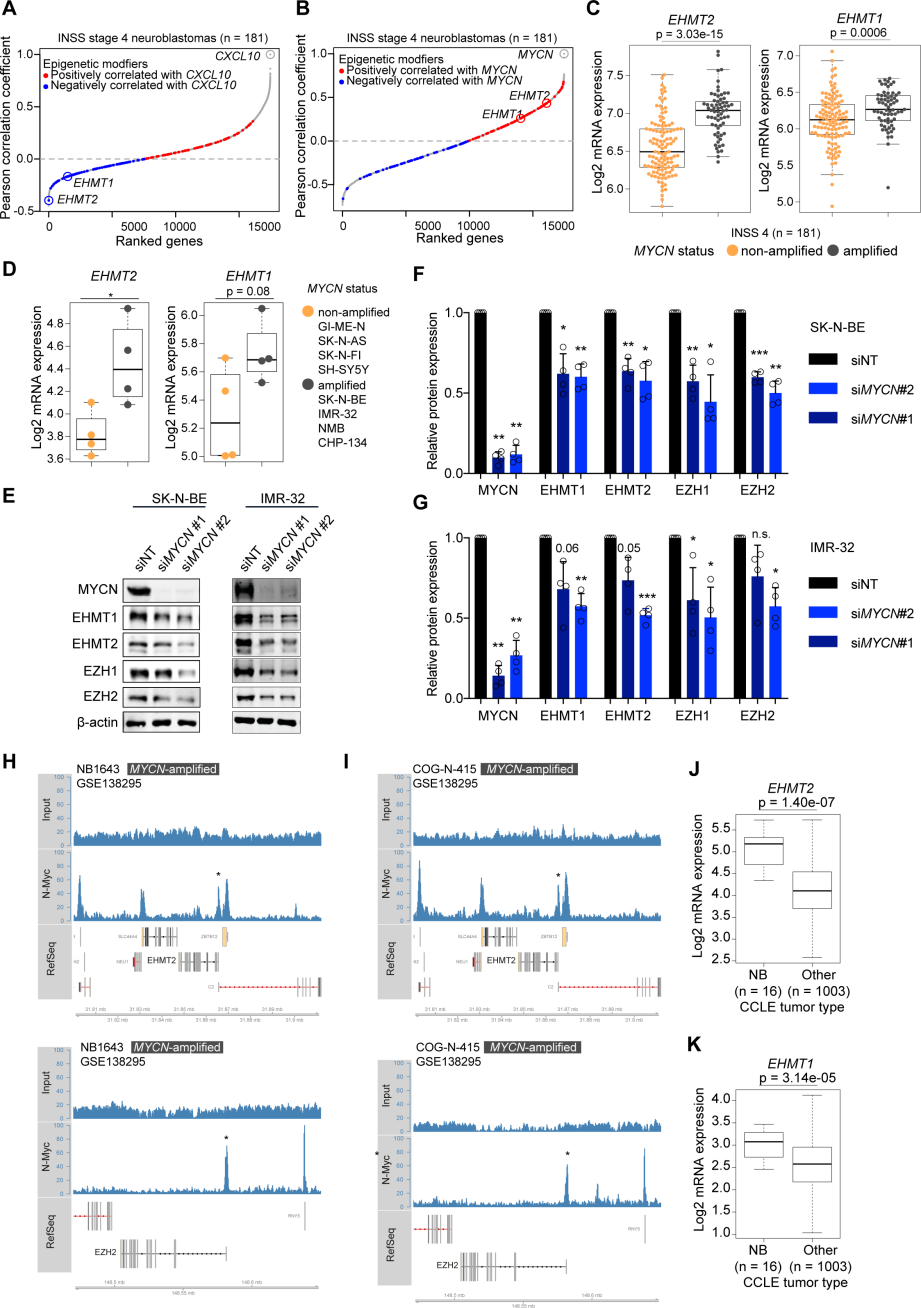
Euchromatic histone methyltransferases (EHMTs) as inversely regulated genes with *CXCL10* level in high-risk neuroblastomas and its association with *MYCN* amplification. (A) Genes correlating with *CXCL10* expression in INSS 4 NBs ranked by increasing Pearson correlation coefficients. Epigenetic modifiers from Xu *et al*^30^ are highlighted. Red: positively correlated; blue: negatively correlated. (B) As in (A) but showing genes correlating with *MYCN* expression. (C) Boxplots showing *EHMT2* and *EHMT1* expression (RNA-seq, log2) in INSS 4 NBs by *MYCN* status. (D) Boxplots showing *EHMT2* and *EHMT1* expression (3′mRNA-seq, log2) in human NB cell lines by *MYCN* status. (E) Western blots for MYCN, EHMT1, EHMT2, EZH1, EZH2 and β-actin in si*MYCN* or non-targeting non-targeting siRNA (siNT)-treated SK-N-BE and IMR-32 cells. Representative blots of biological replicates (n=4). (F, G) Quantification of experiment described in (E) from biological replicates (n=4). Error bars; SD. (H) and (I) MYCN-binding peaks in the genomic regions of *EHMT2* (upper panels) and EZH2 (lower panel) by ChIP-seq. Cell lines and data set as indicated. (J) and (K) *EHMT2* and *EHMT1* expression in NB cells versus other cancer cells from the CCLE database. Statistics: two-sided unpaired t-tests (C, D, J, K). Two-sided unpaired t-test with logarithmic values (F, G). Boxplots: Boxes indicate second and third quartile. Bars indicate first and fourth quartile. Horizontal line represents median. *p<0.05; **p<0.01; ***p<0.001. Otherwise p values as indicated. NB, neuroblastoma.

Previously, we found that depletion of MYCN by RNA interference (RNAi) in NB cell lines enhanced IFN pathway activity, both at baseline and in response to IFN-γ or a synthetic STING agonist.^12^ Now, we asked whether we could identify pharmacological strategies to achieve the same effect. We hypothesized that epigenetic modifiers could be downstream effectors of MYCN and attractive drug targets to restore IFN responsiveness and chemokine expression in NBs.^30^ We therefore analyzed transcriptome data of a well-characterized cohort of INSS stage 4 NBs (metastatic disease) and we ranked genes based on their correlation with the IFN-γ-responsive chemokine *CXCL10*.^31^ Of note, one of the most negatively correlated gene encoded for the euchromatic histone lysine methyltransferase 2 (EHMT2), also known as G9a (figure 1A and online supplemental figure 1A, B). *EHMT1 (GLP*), another histone methyltransferase and closely related to *EHMT2*, was also among the negatively correlated genes with *CXCL10*. EHMT2 and EHMT1 are known to form a complex (G9a/GLP complex) involved in transcriptional silencing by introducing repressive H3K9me2 histone marks.^32 33^ Both *EHMT2* and *EHMT1* mRNA levels were positively correlated with *MYCN* expression (figure 1B) and significantly associated with *MYCN* amplification, in particular *EHMT2* (figure 1C). Transcriptome profiling of our small NB cell line panel (n=8) confirmed higher *EHMT2* expression in *MYCN*-amplified cells (figure 1D). Differences in expression of MYCN and MYC in our NB cell line panel were confirmed by western blot in line with literature (online supplemental figure 1C).^28^ Depletion of MYCN by RNAi moderately reduced the levels of EHMT2 and EZH2 but also EHMT1 and EZH1 (figure 1E–G). The extent of EHMT2 regulation by MYCN was in line with a recent study.^34^ EZH2, a H3K27me3 histone methyltransferase, was previously shown to be a MYCN target conferring epigenetic dependency.^35^ Analyzing publicly available MYCN ChIP-seq data,^28^ we found a MYCN-binding peak in the region of the transcriptional start site of *EHMT2* and confirmed MYCN binding to the *EZH2* promoter as reported (figure 1H, I).^35^ In *MYCN*-non-amplified cells with high MYC expression, MYC peaks in these regions were found variable (online supplemental figure 2A–F). No prominent peaks were detected for MYCN or MYC within the *EHMT1* genomic region (online supplemental figure 2G, H). Together, the results suggested that MYCN, and likely also MYC if abundantly expressed, directly contribute to the expression of both *EHMT2* and *EZH2*. Having addressed the regulation, we next interrogated a cancer type-dependent context of *EHMT* expression. Analysis of the CCLE data set showed that *EHMT2* and *EHMT1* mRNA expression was significantly higher in NB cells when compared with other cell lines from various types of cancers, which indicated potential lineage-specific functions of EHMTs (figure 1J, K).^29^ Altogether, our correlation analyses suggested a potential role for EHMT2 and EHMT1 in the repression of IFN-γ-responsive chemokines like *CXCL10* in NB cells, whereby MYCN and possibly also high levels of MYC directly promote *EHMT2* expression together with *EZH2*.

10.1136/jitc-2020-001335.supp1 Supplementary data

**Figure 2:**
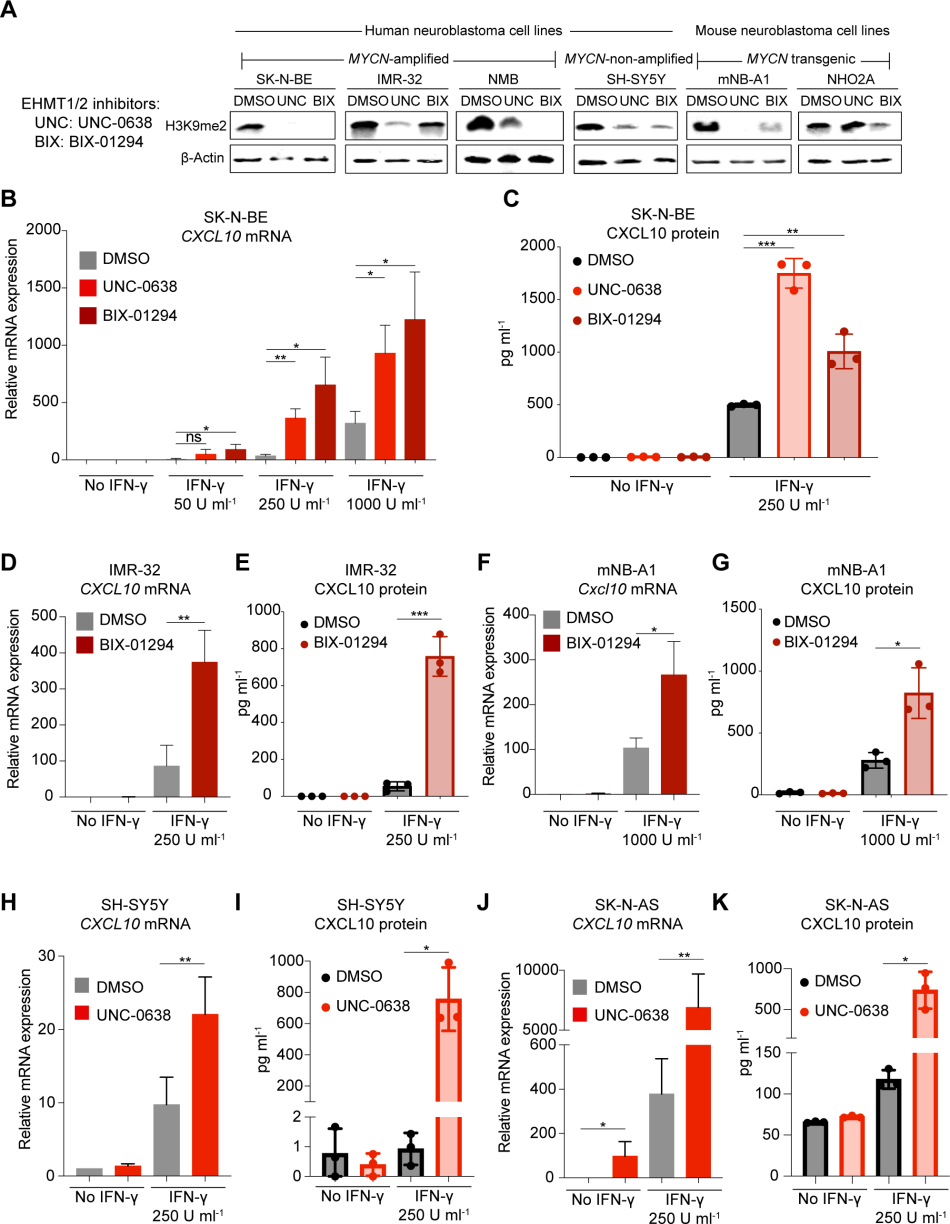
Euchromatic histone-lysine methyltransferase (EHMT) inhibitors enhance IFN-γ-induced CXCL10 production in human and mouse neuroblastoma cell lines. (A) Western blots for H3K9me2 and β-actin of human and mouse NB cell lines treated with EHMT inhibitors UNC-0638 and BIX-01294 for 96 hours (2 µM). Representative blots of n=3. (B) qRT-PCR analysis *CXCL10* mRNA expression (normalized to *UBC*) in SK-N-BE cells treated with UNC-0638 or BIX-01294 for 96 hours (2 µM) and stimulated with increasing concentrations of IFN-γ for the last 24 hours. Results from biological replicates (n=3). (C) Experimental setup as described in (B) but ELISA for CXCL10 protein level (pg mL^−1^) in culture supernatant. Results from biological replicates (n=3). (D) qRT-PCR analysis of *CXCL10* mRNA expression (normalized to *UBC*) and (E) ELISA for CXCL10 protein level (pg mL^−1^) in supernatant. IMR-32 cells treated with BIX-01294 for 96 hours and stimulated with IFN-γ (250 U mL^−1^) for the last 24 hours. Results from biological replicates (n=3). (F, G) as described in (D, E), but mNB-A1 cells. (H, I) and (J, K) as described in (D, E), but SH-SY5Y and SK-N-AS cells. Results from biological replicates (n=3). Statistics: *p<0.05; **p<0.01; ***p<0.001; two-sided unpaired t-test. Error bars: mean±SD. IFN, interferon; NB, neuroblastoma.

To test this hypothesis, we treated four human (SK-N-BE, IMR-32, NMB, SH-SY5Y) and two mouse NB cell lines derived from different *MYCN*-driven genetic models (mNB-A1, NHO2A)^22 23^ with UNC-0638 (UNC) or BIX-01294 (BIX), two selective and well-characterized EHMT inhibitors.^36 37^ As an on-target readout, we confirmed a reduction of H3K9me2 levels on drug exposure by western blot, although variability was noted across the cell lines tested (figure 2A). Both UNC and BIX enhanced IFN-γ-induced expression of *CXCL10* mRNA and protein in *MYCN*-amplified SK-N-BE cells (figure 2B, C). We also confirmed this result in an independent *MYCN*-amplified human NB cell line IMR-32 (figure 2D, E) and the mouse *MYCN*-transgenic NB cell line mNB-A1 (figure 2F, G). In summary, these results supported the idea that EHMTs are involved in the repression of IFN-γ-responsive chemokines associated with high MYCN level in human and mouse NB cells. We also noted that EHMT inhibition enhanced IFN-γ-induced expression of *CXCL10* mRNA and protein in *MYCN* non-amplified SH-SY5Y and SK-N-AS cells, both expressing high levels of MYC (figure 2H–K and online supplemental figure 1C). EHMT inhibitor alone induced *CXCL10* mRNA expression in SK-N-AS cells (figure 2J).

**Figure 3:**
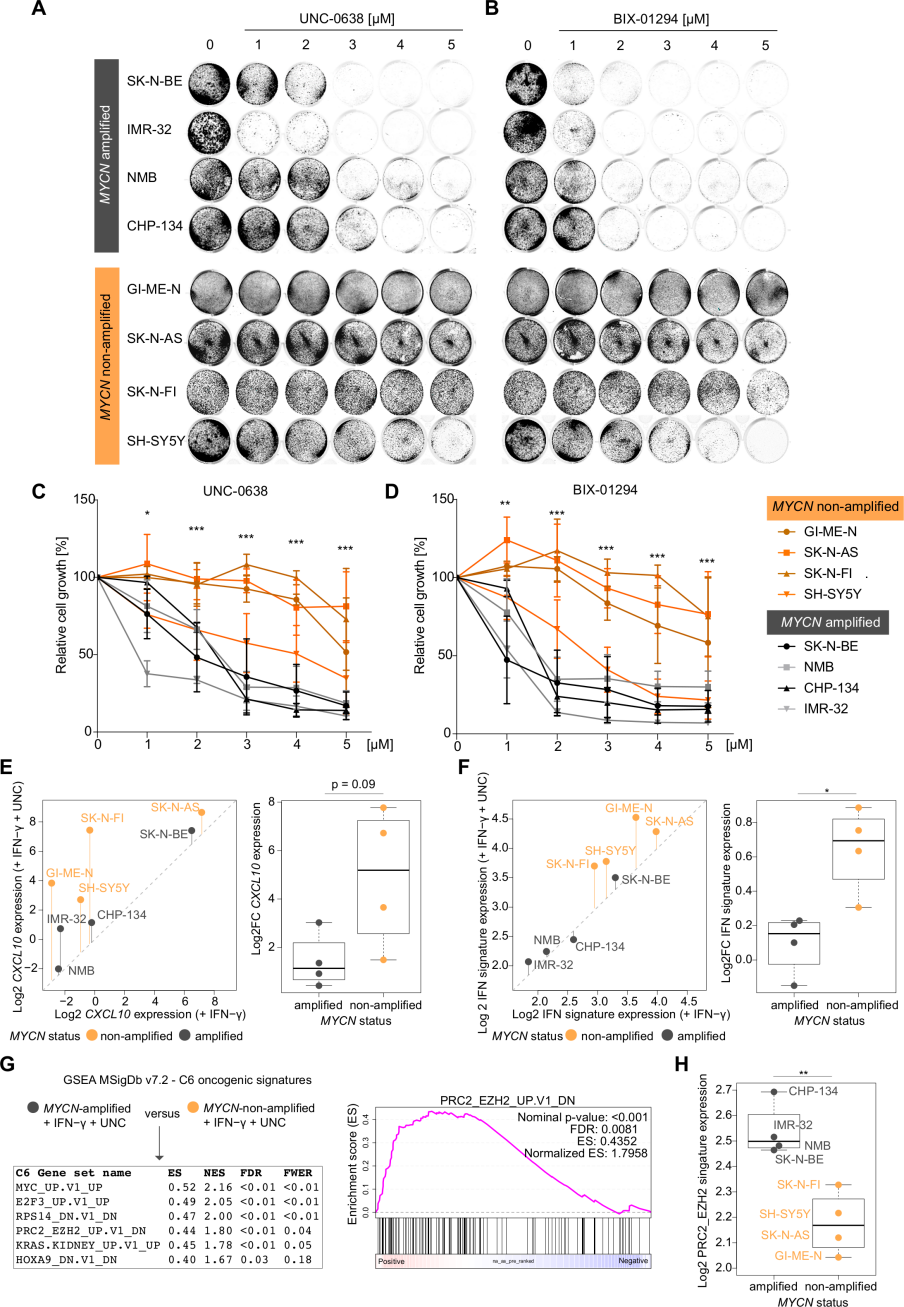
Discordant effects of euchromatic histone-lysine methyltransferase (EHMT) inhibitors on growth inhibition and IFN-γ transcriptional responses dependent on *MYCN* status in human neuroblastoma cells (A) and (B) representative images from biological replicates (n=3) of stained culture dishes of *MYCN*-amplified and *MYCN*-non-amplified human NB cell lines treated with UNC-0638 and BIX-01249 at indicated concentrations for 96 hours. (C) and (D) Quantifications of the experiments described in (A) and (B). (E) *CXCL10* and (F) hallmark interferon gamma response signature expression (log2) in human NB cells based on 3′mRNA-seq data and averaged values from biological duplicates. Left panels: Scatter plots comparing expressing expression in the presence of IFN-γ versus IFN-γ and UNC-0638. Vertical bars indicate log2 fold changes (Log2FCs). Right panels: Log2FCs replotted from scatter plots for statistical comparison. (G) Left panel: GSEA results from group comparison as indicated based on 3′mRNA-seq data described in (E) and (F). Right panel: GSEA plot for indicated gene set. (H) Log2 averaged expression gene set from (G) in NB cell lines stratified by *MYCN* status. Cell lines as indicated. Statistics: *p<0.05; **p<0.01; ***p<0.001; two-sided unpaired t-test with logarithmic relative growth (%) values comparing groups *MYCN*-amplified versus *MYCN*-non-amplified at each concentration in (C) and (D). Two-sided unpaired ratio t-test in (E, F, H). Error bars: mean±SD. Boxplots: Boxes indicate second and third quartile. Bars indicate first and fourth quartile. Horizontal line represents median. ES, enrichments score; FDR, false discovery rate; FWER, family wise error rate; GSEA, gene set enrichment analysis; IFN, interferon; NB, neuroblastoma; NES, normalized ES.

Next, we asked whether EHMT inhibitors also affected the proliferation of NB cells. To this end, we analyzed our panel of four *MYCN*-amplified (SK-N-BE, IMR-32, NMB, CHP-134) and four *MYCN*-non-amplified human NB cell lines (GI-ME-N, SK-N-AS, SK-N-FI, SH-SY5Y) that were exposed to increasing concentrations of UNC or BIX (figure 3A, B). We found that EHMT inhibitors strongly impaired the proliferation of *MYCN*-amplified NB cells, but had a moderate effect on the proliferation of the *MYCN*-non-amplified NB cell lines tested besides SH-SY5Y cells (figure 3C, D). Thus, proliferation of *MYCN*-amplified NB cells was dependent on the activity of EHMTs as also reported by others very recently.^34^ This prompted us to ask whether EHMT inhibition also had concordant effects on IFN-γ responsiveness. We treated each cell line with IFN-γ or IFN-γ plus UNC in biological replicates and analyzed transcriptome changes by 3′mRNA-seq. Interestingly, EHMT inhibition enhanced IFN-γ-induced expression of *CXCL10* and interferon responsive genes (MSigDb Hallmark IFN-γ response) to a larger extent in *MYCN*-non-amplified than in *MYCN*-amplified NB cell lines (figure 3E, F). Actually, global IFN-γ responsiveness on EHMT inhibition of *MYCN*-amplified NB cell lines was rather moderate. In order to analyze the contribution of other oncogenic pathways involved in epigenetic gene silencing, we compared the transcriptomes of the NB cells treated with IFN-γ plus UNC by GSEA using the MSigDb C6 oncogenic signatures collection. Notably, among the significantly (FDR<0.05) regulated gene sets, PRC2_EZH2-UP.V1_DN was enriched in *MYCN*-amplified versus *MYCN*-non-amplified NB cells, which indicated higher EZH2 activity in the former (figure 3G, H). Together with embryonic ectoderm development protein (EED) and SUZ12 polycomb repressive complex 2 (PRC2) subunit (SUZ12), EZH2 (as well as EZH1) is a core component of PRC2 involved in gene silencing. Of note, PRC2 physically interacts with the EHMT (G9a/GLP) complex and functionally cooperates in transcriptional repression.^33^ Thus, GSEA suggested to combine PRC2 inhibitors (eg, EZH2 inhibitors) with EHMT inhibitors in order to achieve robust transcriptional responses to IFN-γ also in *MYCN*-amplified NB cells.

**Figure 4:**
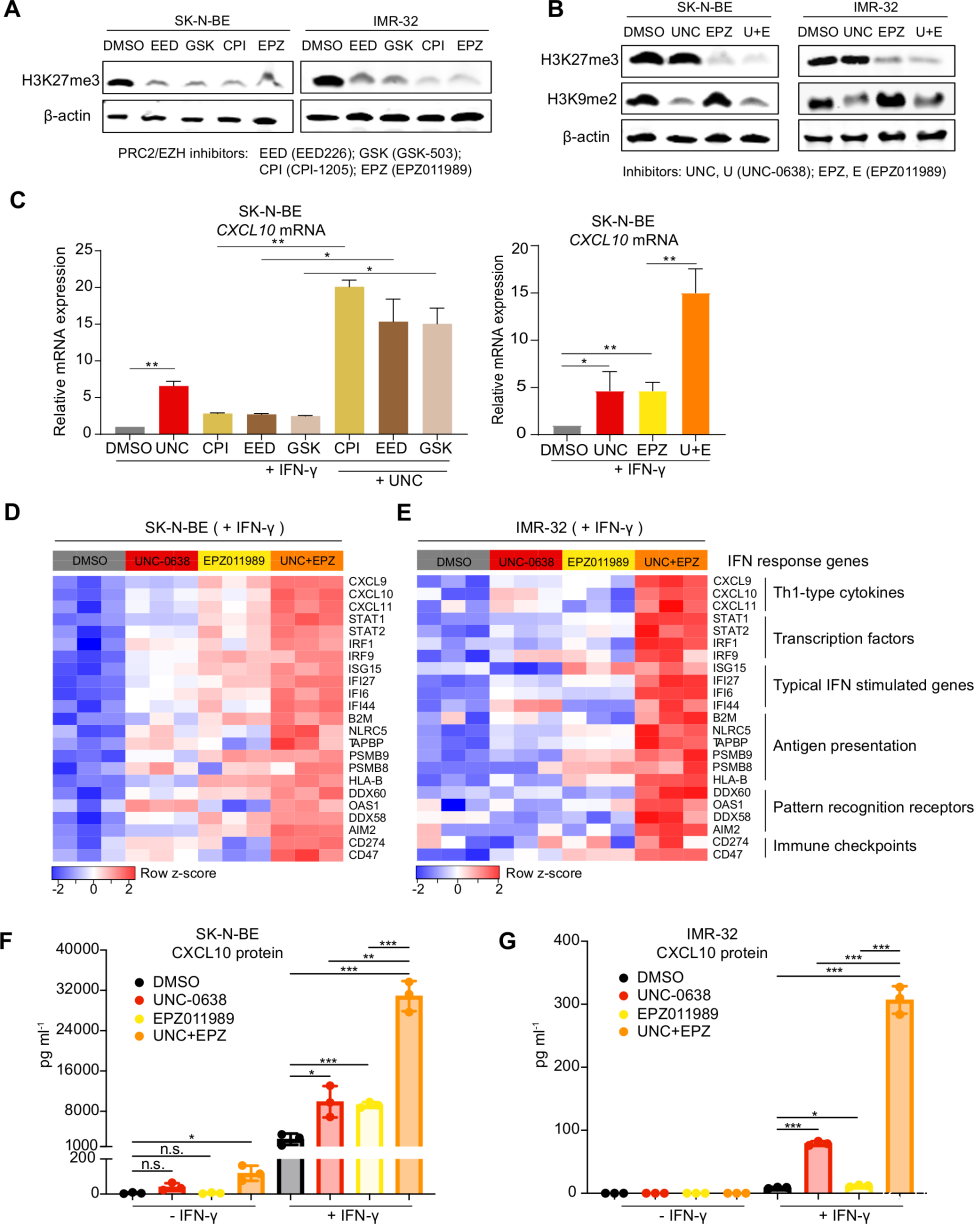
Combined euchromatic histone-lysine methyltransferase (EHMT) and EZH2 inhibition restores robust transcriptional responses to IFN-γ and CXCL10 chemokine production in *MYCN*-amplified human neuroblastoma cells. (A) Western blots H3K27me3 and β-actin in SK-N-BE and IMR-32 cells treated with different PRC2 and EZH2 inhibitors (all 3 µM) for 96 hours. Representative blots of n=3. (B) Western blots for H3K27me3, H3K9me2 and β-actin in SK-N-BE and IMR-32 cells treated with EHMT inhibitor UNC-0638 (2 µM), EZH2 inhibitor EPZ011989 (3 µM) or the combination of both drugs for 96 hours. Representative blots of n=3. (C) *CXCL10* mRNA expression assessed by qRT-PCR in SK-N-BE NB cells treated as indicated. Results from biological replicates (n=3). (D) Heatmap visualizing the transcriptional response to IFN-γ in SK-N-BE and (E) IMR-32 cells treated with vehicle or UNC-0638, EPZ011989 or both for 7 days. IFN-γ (250 U/mL) was added for the last 24 hours. Experiments performed in biological replicates (n=3). (F, G) ELISA for CXCL10 protein level (pg/mL) in supernatants from SK-N-BE (F) and IMR-32 NB (G) cells treated as described in (C) and (D). Results from biological replicates (n=3). Statistics: *p<0.05; **p<0.01; ***p<0.001; two-sided unpaired t-test. Error bars: mean±SD. IFN, interferon; NB, neuroblastoma.

To test this idea, we treated SK-N-BE and IMR-32 cells with the EED inhibitor EED226 (EED) or the EZH inhibitors GSK-503 (GSK), CPI-1205 (CPI) or EPZ011989 (EPZ) and confirmed on-target reduction of H3K27me3 levels by western blot analysis (figure 4A). All three EZH inhibitors are more effective against EZH2 than EZH1.^36^ To test combined effects, we used UNC-0638 (UNC) and EPZ011989 (EPZ). At least in the time frame analyzed, UNC reduced H3K9me2 but not H3K27me3 levels, and vice versa EPZ, confirming on-target activity of the inhibitors (figure 4B). NB cells treated with a combination of both drugs (UNC+EPZ) consistently showed reduced levels of both histone methylation marks. In combination with EHMT inhibition by UNC, all EZH or EED inhibitors enhanced IFN-γ-induced *CXCL10* mRNA expression (figure 4C). Next, we performed 3′mRNA-seq transcriptome analysis of SK-N-BE and IMR-32 cells in the absence or presence of IFN-γ, in addition to treatment with vehicle, UNC, EPZ or both drugs (UNC+EPZ). Single drug treatment with either UNC or EPZ enhanced the transcriptional response to IFN-γ, but the combination treatment UNC+EPZ was more effective in both NB cell lines (figure 4D, E). Effects were not restricted to IFN-γ-inducible Th1-type chemokines *CXCL9*, *CXCL10* or *CXCL11*, but included typical IFN-γ-inducible genes from various functional categories such as transcription factors (eg, *STAT1*, *IRF1*), antigen presentation (*B2M*, *NLRC5*, *PSMB9*, *HLA-B*), pattern recognition receptors (*DDX58* encoding RIG-I) and immune checkpoints (*CD274* encoding PD-L1). The combination treatment UNC+EPZ also strongly enhanced IFN-γ-induced CXCL10 release into cell culture supernatants assessed by ELISA (figure 4F, G).

**Figure 5:**
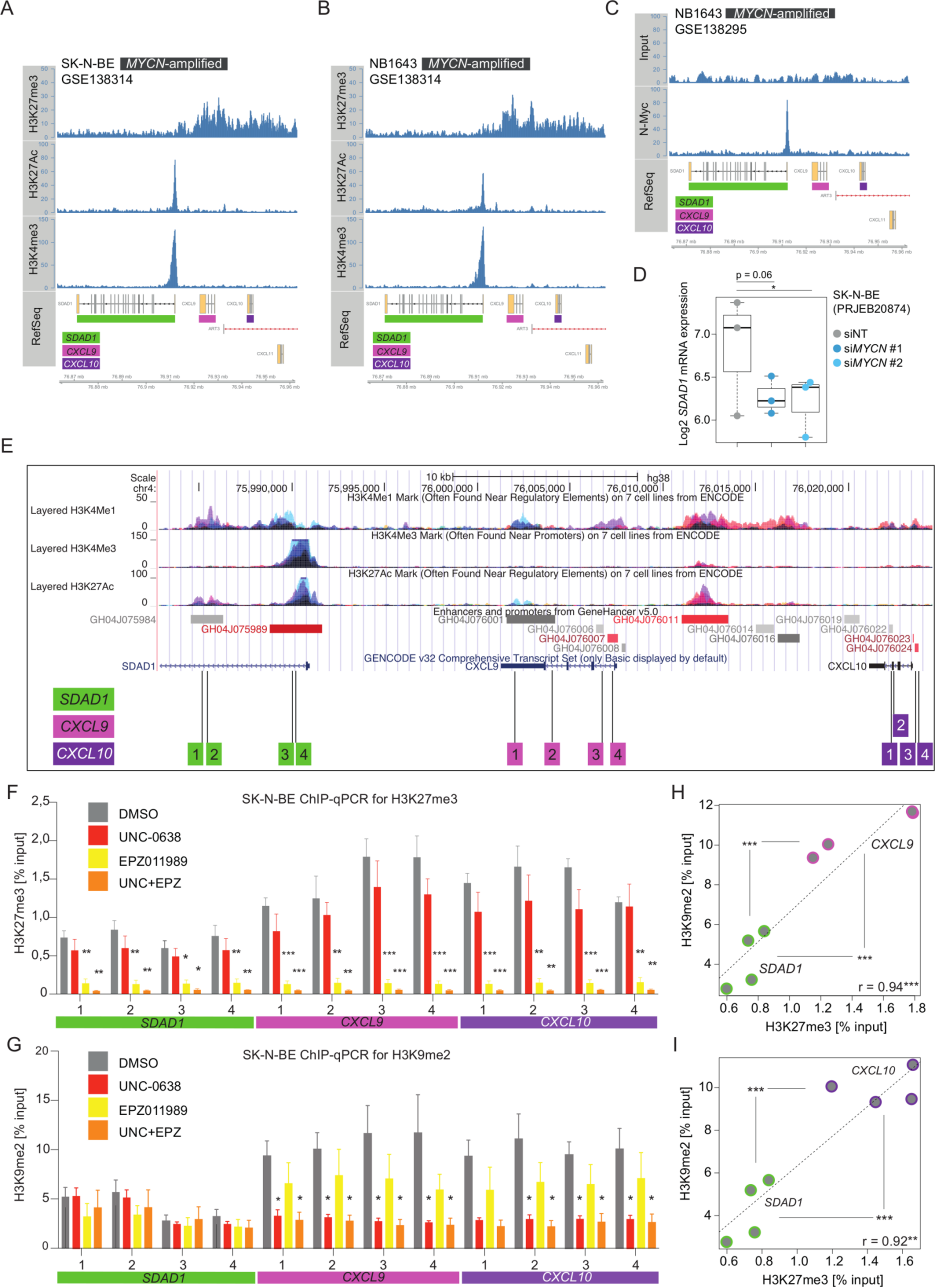
Loss of H3K9me2 and H3K27me3 repressive histone marks at *CXCL9* and *CXCL10* genomic loci on euchromatic histone-lysine methyltransferase (EHMT) and EZH2 inhibitor treatment of human neuroblastoma cells. Plotted histone ChiP-seq tracks obtained GSE138314 from (A) SK-N-BE and (B) NB1643 *MYCN*-amplified cells showing the genomic region of *CXCL9*, *CXCL10* and *CXCL11* chemokines genes with neighboring *SDAD1* gene on human chromosome 4. (C) As (B), but showing MYCN-binding ChIP-seq track and input obtained from GSE138295. (D) *SDAD1* expression (3′mRNA-seq, log2, from PRJEB20874) in SK-N-BE cells transfected with non-targeting siRNA (siNT) and MYCN siRNAs. (E) UCSC genome browser plot showing the genomic regions of *SDAD1*, *CXCL9*, *CXCL10* and *CXCL11* with ENCODE ChIP-seq tracks. Strategy of PCR primer pair positioning for tiling ChIP-qPCR in regulatory region of respective genes. Results from biological replicates (n=3) from tiling ChIP-qPCR for (F) H3K27me3 and (G) H3K9me2 represented as percentage input. SK-N-BE cells were treated with indicated inhibitors for 6 days prior to harvesting chromatin. Numbers on x-axis represent primer pairs as described in (E). (H) Scatter plot comparing and correlating baseline level of H3K27me3 and H3K9me2 as (% input) between neighboring *SDAD1* and *CXCL9* genomic regions based on results from (F) and (G) in untreated SK-N-BE cells. Statistically significant differences between groups (*SDAD1* and *CXCL9*) are indicated. (I) Same analysis as in (H), but for *CXCL10*. Statistics: *p<0.05; **p<0.01; ***p<0.001; two-sided unpaired t-tests with logarithms of percentage input values; p values corrected for multiple testing with Benjamini and Hochberg method (FDR) in (E) and (G). Error bars: mean±SEM (F, G). Boxplots: boxes indicate second and third quartile. Bars indicate first and fourth quartile. Horizontal line represents median.

Next, we addressed the epigenetic context how UNC+EPZ promotes IFN-γ responses. In particular, we were interested in the *CXCL9*, *CXCL10* and *CXCL11* Th1-type chemokines clustered on chromosome 4. We therefore analyzed published histone ChIP-seq data that provided epigenomic profiling of several NB cell lines, both *MYCN*-amplified and *MYCN*-non-amplified.^28^ The data set included ChIP-seq for the repressive histone mark H3K27me3 as well as for activating histone marks (H3K27Ac, H3K4me3), whereas H3K9me2 was not assessed in this study. We found enriched H3K27me3 signals in the entire *CXCL9*, *CXCL10* and *CXCL11* genomic region in five out of six *MYCN*-amplified NB cell lines (SK-N-BE, NB1643, COG-N415, NGP, Kelly and LAN-5) (figure 5A, B and online supplemental figure 3A–D), but only in one out of four *MYCN*-non-amplified NB cell lines (NB-69, NBLS, SK-FI, SK-N-AS) (online supplemental figure 3E-H). H3K27me3 signals were low in the region of the neighboring gene *SDAD1*, which in contrast showed prominent ChIP-seq peaks of the activating histone marks H3K27ac and H3K4me3 in the promotor region (figure 5A, B and online supplemental figure 3A–D). Further, *SDAD1* showed a prominent MYCN-binding beak in its promoter region (figure 5C), and previous transcriptome profiling of MYCN-depleted NB cells by RNAi suggested that *SDAD1* is a potential MYCN target gene (figure 5D). Importantly, no MYCN-binding or MYC-binding peaks were found in the genomic region of *CXCL9*, *CXCL10* and *CXCL11* (figure 5D and online supplemental figure 3I). Recently, MYC was found to directly interact with EHMTs mediating gene repression and promoting tumorigenesis.^38^ At least in the case of regulation of *CXCL9*, *CXCL10* and *CXCL11* expression in NB cells, neither MYCN nor MYC seemed to be directly involved in the recruitment of the EHMT/EZH2 repressive complexes, but indirectly by increasing the levels of EHMT2 and EZH2 in NB cells. As we had also profiled IFN-γ responses of two *MYCN*-non-amplified NB cell lines SK-N-FI and SK-N-AS (high vs low H3K27me3 signals at *CXCL9*, *CXCL10* and *CXCL11* loci) overlapping with the ChIP-seq study,^28^ we consistently noticed that low H3K27me3 signals in SK-N-AS cells correlated with strong *CXCL9*, *CXCL10* and *CXCL11* gene induction by IFN-γ treatment alone (online supplemental figure 3G, H, J).

Given that neither H3K9me2 was profiled in Upton *et al.*^28^ nor exposures to EZH2 or EHMT inhibitors were tested, we performed ChIP-qPCRs for H3K27me3 and H3K9me2 assessing regulatory regions within *CXCL9*, *CXCL10* and *SDAD1* as control at four different genomic sites each (figure 5E). Control ChIP-qPCRs for histone H3 are shown in online supplemental figure 4. SK-N-BE cells were treated with UNC, EPZ or UNC+EPZ for 6 days in biological triplicates and chromatin was harvested for ChIP-qPCR analyses. First, H3K27me3 level (% input) was higher at all regions in *CXCL9* and *CXCL10* compared with *SDAD1* in untreated cells (figure 5F). Exposure to EPZ or UNC+EPZ resulted in a strong reduction of H3K27me3 level at all loci reaching a similar baseline level (figure 5F). Consistently, H3K9me2 level (%input) was also higher at all tested regions in *CXCL9* and *CXCL10* compared with the *SDAD1* control regions in untreated cells (figure 5G). Notably, UNC or UNC+EPZ treatment resulted in a reduction of H3K9me2 level in all tested regions in *CXCL9* and *CXCL10*, but regions in *SDAD1* remained unaltered (figure 5G). Furthermore, H3K9me2 and H3K27me3 level closely correlated at regions in *CXCL9* and *CXCL10* underscoring the importance of both repressive histone marks in the regulation of *CXCL9* and *CXCL10* chemokine gene expression (figure 5H, I).

**Figure 6:**
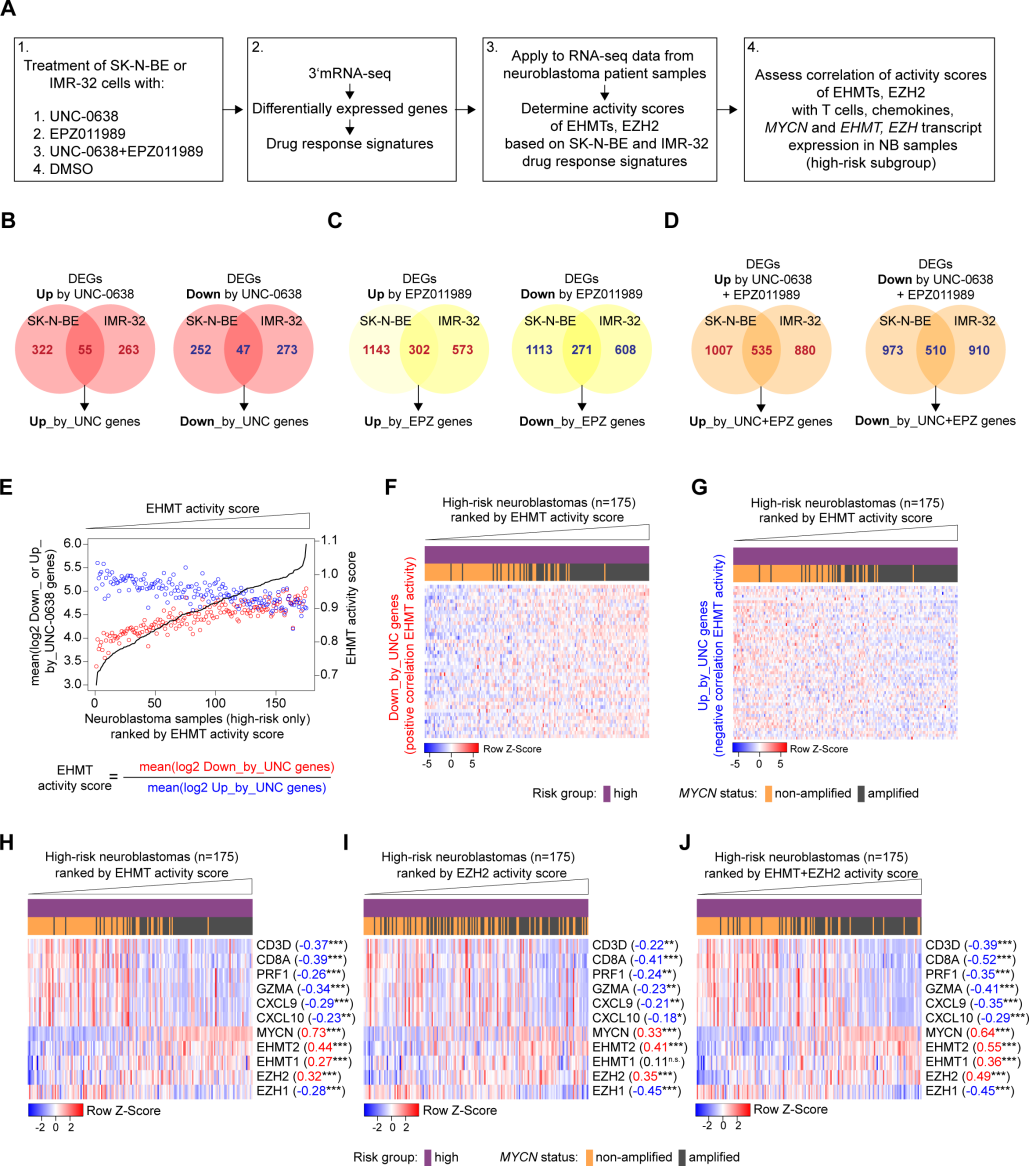
High-risk neuroblastomas with high euchromatic histone-lysine methyltransferase (EHMT) and EZH2 activity are characterized by *MYCN* amplification and a T-cell infiltration-poor tumor microenvironment. (A) Outline of bioinformatic strategy. (B, C, D) Generation of drug response signatures from overlap of differentially expressed genes in SK-N-BE and IMR-32 cells treated with UNC-0638 (B), EPZ011989 (C) or both drugs (D). (E) Exemplary visualization of calculation of EHMT activity scores for high-risk NB samples. (F, G) Exemplary heatmap visualization of expression of UNC-0638 drug response genes in high-risk NB samples ranked by increasing EHMT activity score. (H–J) Heatmaps visualizing immune contexture marker genes (eg, *CD8A*, *CXCL10*), *EHMT2/1*, *EZH2/1* and *MYCN* in high-risk NB samples ranked by increasing activity scores of EHMT (H), EZH2 (I) and EHMT+EZH2 (J). Pearson correlation coefficients are indicated besides the names of the transcripts. Statistics: *p<0.05; **p<0.01; ***p<0.001; two-sided t-test for Pearson product moment correlation coefficient. NB, neuroblastoma.

Next, we aimed to establish UNC and EPZ drug response gene signatures from NB cell lines as surrogate measures of EHMT and EZH2 activity for the analysis of transcriptomes from NB patient samples (figure 6A). We reasoned that expression level of EHMTs or EZH2 might or might not correlate with EHMT and EZH2 activity in NB patient samples and drug response signatures could be better estimates of activity. We also took into consideration that EHMT and EZH2 inhibitors have both direct and indirect effects on transcriptional responses, for which reason we included both upregulated and downregulated genes into our analysis.^27^ Thus, we treated SK-N-BE and IMR-32 NB cells with UNC, EPZ or both drugs and performed 3′mRNA-seq as described above, without IFN-γ exposure. We then determined the differentially expressed genes (DEGs) that were either upregulated or downregulated (FDR<0.05) by UNC, EPZ or the combination drug exposure UNC+EPZ in SK-N-BE and IMR-32 cells (online supplemental tables S1–S3). Consequently, we obtained six drug response signatures, for example, ‘Up_by_UNC-0638’ and ‘Down_by_UNC-0638’, based on the overlap of the DEGs (figure 6B–D and online supplemental tables S4–S6). Of note, *MYCN* was 1 of the 47 genes in the ‘Down_by_UNC-0638’ signature. However, *MYCN* was omitted from downstream analysis because *MYCN* expression is known to strongly stratify NB samples. In order to determine surrogate EHMT activity scores in transcriptomes from NB patient samples, we divided the averaged log2 expression values of ‘Down_by_UNC-0638’ genes (positive correlation with EHMT activity) by the averaged log2 expression values of ‘Up_by_UNC-0638’ genes (negative correlation with EHMT activity) for each NB sample (figure 6E). Thus, a higher score would indicate a higher EHMT activity. Heatmaps visualize opposing expression pattern of ‘Down_by_UNC-0638’ genes and ‘Up_by_UNC-0638’ genes in high-risk NBs ranked by increasing EHMT activity scores (figure 6F, G). Scores for surrogate EZH2 activity and combined EHMT+EZH2 activity were calculated in the same manner as EHMT activity scores. EPZ is rather selective for EZH2, and therefore, EZH activity scores were assumed to reflect EZH2 activity.^39^

10.1136/jitc-2020-001335.supp2 Supplementary data

10.1136/jitc-2020-001335.supp3 Supplementary data

10.1136/jitc-2020-001335.supp4 Supplementary data

10.1136/jitc-2020-001335.supp5 Supplementary data

10.1136/jitc-2020-001335.supp6 Supplementary data

10.1136/jitc-2020-001335.supp7 Supplementary data

**Figure 7:**
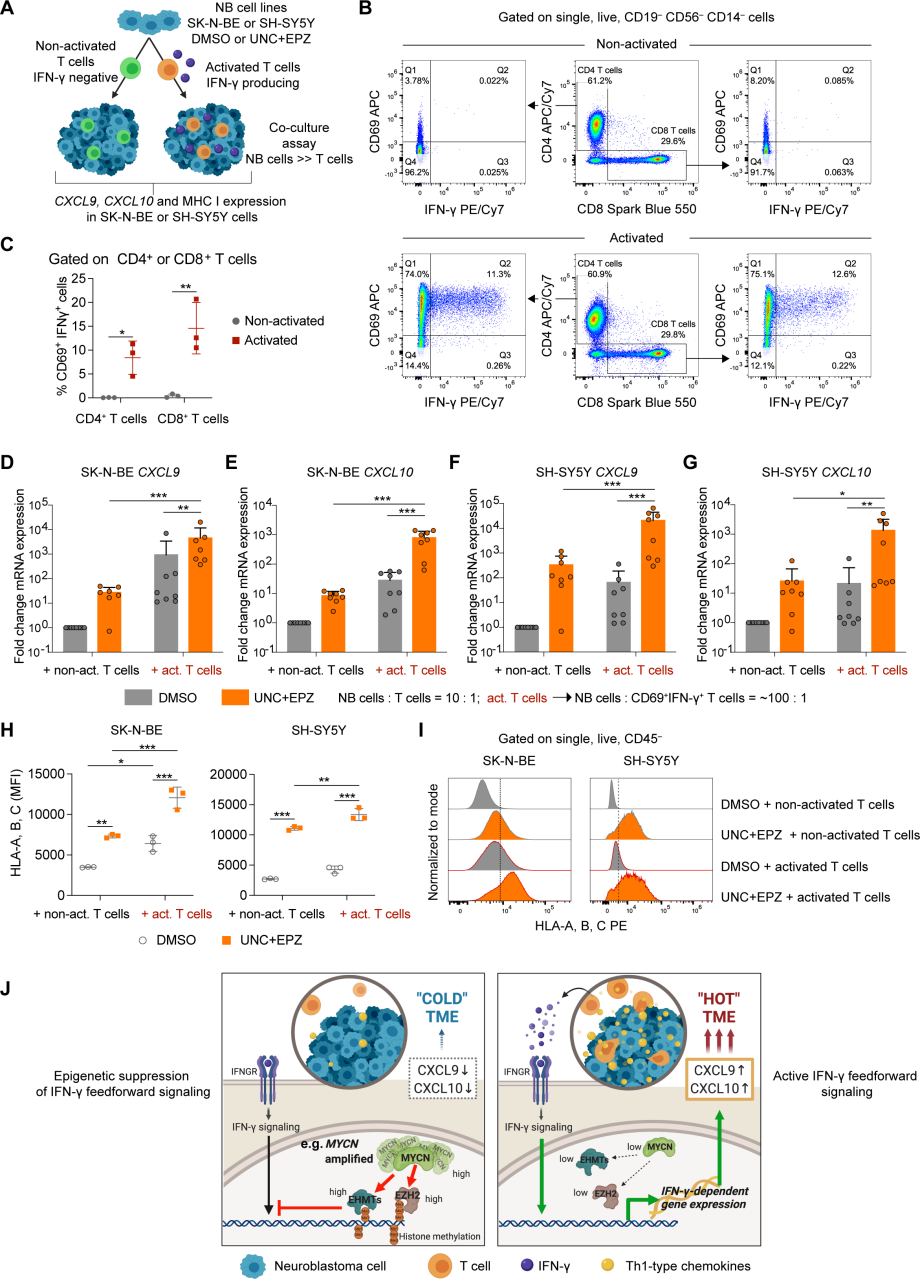
Combined euchromatic histone-lysine methyltransferase (EHMT) and EZH2 inhibition amplifies chemokine and MHC I expression by neuroblastomas instigated by low frequency of activated IFN-γ producing T cells. (A) Outline of experimental strategy. (B) Gating strategy and flow cytometric detection of activated CD69^+^IFN-γ^+^ human T cells after treatment with anti-CD3/CD28. (C) Quantification of frequency of CD69^+^IFN-γ^+^ T cells from three independent donors. (D–G) *CXCL9* and *CXCL10* mRNA expression by qRT-PCR in SK-N-BE (D, E) and SH-SY5Y (F, G) cells co-cultured and treated as indicated. (H, I) Flow cytometric analysis of MHC I expression on SK-N-BE and SH-SY5Y cells co-cultured and treated as indicated. (J) Model summarizing our findings. Statistics: two-way ANOVA with multiple comparison (C, H). Two-sided unpaired t-test with logarithmic values (D–G). Horizontal line represents median. *P<0.05; **p<0.01; ***p<0.001. Otherwise p values as indicated. IFN, interferon.

In high-risk NB samples, *EHMT2, EHMT1* and *MYCN* mRNA levels were positively correlated with the EHMT activity score with the highest values observed for *EHMT2* and *MYCN*, respectively (figure 6H). Interestingly, EHMT activity score was negatively correlated with surrogate cytotoxic T-cell content (*CD8A, PRF1, GZMA*) and Th1-type chemokines (*CXCL9*, *CXCL10*) further supporting a link between high EHMT activity and a ‘cold’ immune phenotype in NB. We repeated the analysis for the EZH2 activity score showing a positive correlation with *EZH2* gene expression, but a negative correlation with *EZH1* gene expression (figure 7I). T-cell content (*CD8A, PRF1, GZMA*) and Th1-type chemokines (*CXCL9*, *CXCL10*) were also negatively correlated with the EZH2 activity score, but weaker than the EHMT activity score. Finally, we repeated the analyses for the combined EHMT+EZH2 activity score, which revealed a positive correlation with *MYCN*, *EHMT2*, *EHMT1* and *EZH2* gene expression (figure 7J). In comparison with individual EHMT or EZH activity scores, the combined EHMT+EZH2 activity score showed the strongest negative correlation with immune contexture marker genes for cytotoxic T cells (*CD8A, PRF1, GZMA*) and Th1-type chemokines (*CXCL9*, *CXCL10*). Of interest, the combined EHMT+EZH2 activity score also showed significant negative correlations with the immune marker genes when restricting the analyses to *MYCN*-amplified cases only (online supplemental figure 5). Together with our experimental data, the bioinformatic analyses suggested that high EHMT and EZH2 activity contribute to the T-cell poor cold immune phenotype of high-risk NBs with *MYCN* amplification providing a rationale for targeted epigenetic immunomodulation.

To mimic this scenario, we devised a functional assay co-culturing a bulk population of UNC+EPZ or vehicle-treated SK-N-BE or SH-SY5Y cells with a low frequency of human T cells as a natural source of IFN-γ rather than adding recombinant IFN-γ (figure 7A). *MYCN*-non-amplified SH-SY5Y cells expressed high levels of MYC (online supplemental figure 1C) and showed the strongest antiproliferative response to EHMT inhibitors among the *MYCN*-non-amplified NB cell lines tested (figure 3A–D), for which reason SH-SY5Y cells were also a valuable model. We decided to compare non-activated with activated T cells (anti-CD3, anti-CD28) in co-cultures, as IFN-γ production would be only detected in the latter. Indeed, flow cytometric analyses confirmed that CD69^+^ IFN-γ^+^ CD4^+^ or CD8^+^ T cells were exclusively detected when T cells were exposed to anti-CD3 and anti-CD28 (figure 7B). On average, about 10% of T cells (CD4^+^ and CD8^+^) were CD69^+^IFN-γ^+^ in the pool of anti-CD3 and anti-CD28-activated T cells (figure 7C). We then decided to co-culture SK-N-BE or SH-SY5Y cells with non-activated or activated T cells at a ratio of 10:1. By this, we obtained a final ratio of SK-N-BE or SH-SY5Y cells to CD69^+^ IFN-γ^+^ T cells of about 100:1 when adding the activated T-cell pool. After co-culture, we analyzed *CXCL9* and *CXCL10* mRNA expression in NB cells showing the strongest induction when SK-N-BE or SH-SY5Y cells were pretreated with UNC+EPZ and co-cultured with activated T cells (figure 7D–G). We also analyzed MHC class I surface expression on SK-N-BE or SH-SY5Y showing again the highest levels when the NB cells were pretreated with UNC+EPZ and co-cultured with activated T cells (figure 7H, I). Thus, a low frequency of CD69^+^IFN-γ^+^ T cells (~1%) strongly promoted *CXCL9* and *CXCL10* chemokine expression and MHC I expression in co-culture assays if the NB cells were pretreated with EHMT and EZH inhibitors. Based on this, we propose that EHMT and EZH inhibitors could instigate a feedforward loop of IFN-γ^+^ and Th1-type chemokine expression in the TME, which would be therapeutically most beneficial in the case of NBs with high EHMT and EZH activity such as *MYCN*-amplified NBs (figure 7J). Nevertheless, also a subset of *MYCN*-non-amplified NBs might benefit from this strategy, but the precise context of high EHMT and EZH2 activity within this subset remains to be determined.

## Discussion

*MYCN* amplification has been previously shown by us and others to be associated with a T-cell poor TME phenotype in metastatic NB.^12 13 40^ As drugging MYCN remains a challenge, our study asked whether epigenetic modifiers could be promising drug targets for immunomodulation of NB. Through the exploration of NB transcriptomes, we identified euchromatic histone lysine methyltransferases EHMT2 and EHMT1 as potential suppressors of IFN-γ responsive Th1-type chemokines like *CXCL10*, which was confirmed experimentally using selective small molecule inhibitors. We also provided evidence for a functional cooperation between EHMTs and EZH2 in the suppression of IFN pathway activity, in line with a report showing that these epigenetic modifiers mediate gene silencing in a concerted manner.^33^ Levels of the repressive histone marks H3K9me2 and H3K27me3 were enriched at the *CXCL9*, C*XCL10* and *CXCL11* chemokine cluster in SK-N-BE cells when compared with neighboring regions and correlated. While our manuscript was in revision, two preprints also propose epigenetic mechanisms, EZH2 and phenotypic cell states, as regulators and determinants of NB immune signaling.^41 42^ Together with our work, this underscores the emerging concept of using epigenetic drugs for targeted immunomodulation of high-risk NBs.

The first functional associations between EHMT activity and IFN signaling were reported in the context of antiviral responses. An early study described that EHMT2 was responsible for silencing of IFN-β (*IFNB1*) gene transcription through a cooperation with the transcription factor PRDM1 (BLIMP-1).^43^ More recent studies have established a role for H3K9me2 in the repression of IFNs and IFN-stimulated genes, which could be overcome by genetic or pharmacological inhibition of EHMT2, for example, in fibroblasts.^44^ With regard to cancer therapy, EHMT inhibition by BIX-01294 has been shown to sensitize chronic myeloid leukemia cells to type I IFNs.^45^ A dual inhibitor (CM-272) of EHMTs and DNA methyltransferases (DNMTs) was demonstrated to be active against multiple preclinical models of hematological neoplasia as well as in enhancing IFN responsiveness.^46^ Additionally, CM-272 also promoted TME phenotype conversion from ‘cold’ to ‘hot’ in a mouse model of bladder cancer synergizing with anti-PD-1 ICB therapy.^47^ Despite these published links between EHMTs and suppression of IFN responses, such a role is yet to be established for NB. Importantly, our study has defined the clinical context in which *EHMT2*, *EHMT1* together with *EZH2* are expressed at high levels, mostly *MYCN*-amplified high-risk NBs with a T-cell-poor and non-inflamed TME phenotype. Our low-frequency T-cell co-culture assays depicted how EHMT and EZH2 inhibition could amplify weak IFN feedforward signaling in the TME of high-risk NBs. We believe that this epigenetic treatment approach could have important implications for the future development of immune checkpoint inhibitor therapy in NB to overcome the current limitations.^17 18^ Our limited data on NB cell lines also suggest that EHMT inhibition alone might be sufficient for MYCN-low and MYC-low NBs, but this would require future in-depth studies.

Although antiproliferative and cytotoxic effects of EHMT inhibitors in *MYCN*-amplified NB cells were reported very recently,^34^ our EHMT activity score further substantiated the notion that EHMTs are effectors of the MYCN-driven malignant phenotype in NB. Hence, exploiting this genotype-dependent vulnerability could be a promising therapeutic strategy for *MYCN*-amplified NBs. Even though our re-analysis of public ChIP-seq data indicated binding of MYC to the regulatory regions of *EHMT2* and *EZH2*, less total MYC versus MYCN and/or functional differences between MYC and MYCN may explain higher expression and dependence on EHMT2 and EZH2 activity in *MYCN*-amplified NB cell lines. A CRISPR-Cas9 screen recently showed that *MYCN*-amplified NB cells were also highly dependent on EZH2 activity,^35^ a finding also supported by our observations whereby the combined EHMT+EZH2 activity score strongly correlated with *EHMTs*, *EZH2* and *MYCN* expression. MYCN has been also shown to physically interact with EZH2-containing PRC2.^48^ Therefore, our work and that of others encourage further exploration of epigenetic drugs for the treatment of NB.^34 35 49–51^

## Conclusion

In summary, our findings establish a link between increased EHMT and EZH2 activity and a T-cell-poor, cold TME immune phenotype in high-risk NB, by focusing on the epigenetic regulation of Th1-type chemokine genes with *CXCL9* and *CXCL10* being two key drivers of T-cell recruitment.^52^ Our experimental data further suggests that EHMTs and EZH2 are causally involved in the repression of interferon signaling, which promotes the establishment of a non-inflamed ‘cold’ TME by interfering with IFN-γ feedforward signaling. Therefore, our study provides a scientific basis to explore EHMT inhibitors, alone (maybe in a subset of *MYCN*-non-amplified NBs) or in combination with EZH2 inhibitors (in particular *MYCN*-amplified NBs), for targeted immunomodulation of NBs to induce a ‘hot’ TME phenotype for improved responsiveness to ICB therapy. Our proposed epigenetic treatment strategy also has the potential to synergize with efforts employing STING agonists or oncolytic viruses for multimodal immunotherapy of NB.^20 53^ Nevertheless, further preclinical work will be needed to delineate optimized drug combinations and regimens that could achieve long-term immunological control of high-risk NB.

## Data Availability

All data relevant to the study are included in the article or uploaded as supplemental information. Raw sequencing data is available through the European Nucleotide Archive (ENA) under the accession numbers PRJEB38901 and PRJEB42679. Other data and source code are available upon request. All data needed to evaluate the conclusions in the paper are present in the paper and/or the online supplemental materials.
