# Supplementary material for: Druggable epigenetic suppression of interferon-induced chemokine expression linked to MYCN amplification in neuroblastoma
Source: J Immunother Cancer. 2021 May 20;9(5):e001335. doi: 10.1136/jitc-2020-001335 (PMC8141444; doi:10.1136/jitc-2020-001335)
Supplement: Supplementary data [file jitc-2020-001335supp001.pdf]

Online supplemental material to

Druggable epigenetic suppression of interferon-induced chemokine expression linked to *MYCN*-amplification in neuroblastoma

Authors

Johanna A. Seier[1], Julia Reinhardt[1], Kritika Saraf[1], Susanna S. Ng[1], Julian P. Layer[1,2], Dillon Corvino[1], Kristina Althoff[3], Frank A. Giordano[2], Alexander Schramm[3], Matthias Fischer[4,5], Michael Hölzel[1,#]

Affiliations

[1]Institute of Experimental Oncology, Medical Faculty, University Hospital Bonn, University of Bonn, 53127 Bonn, Germany

[2]Department of Radiation Oncology, University Hospital Bonn, University of Bonn, Bonn, Germany

[3]Department of Medical Oncology, West German Cancer Center, University Hospital Essen, University of Duisburg-Essen, 45147 Essen, Germany.

[4]Department of Experimental Pediatric Oncology, University Children's Hospital of Cologne, Faculty of Medicine and University Hospital of Cologne, Kerpener Straße 62, 50937 Cologne, Germany.

[5]Center for Molecular Medicine Cologne (CMMC), University of Cologne, Robert-Koch-Straße 21, 50931 Cologne, Germany.

[#]Corresponding author: michael.hoelzel@ukbonn.de

Seier et al. online supplemental figure 1

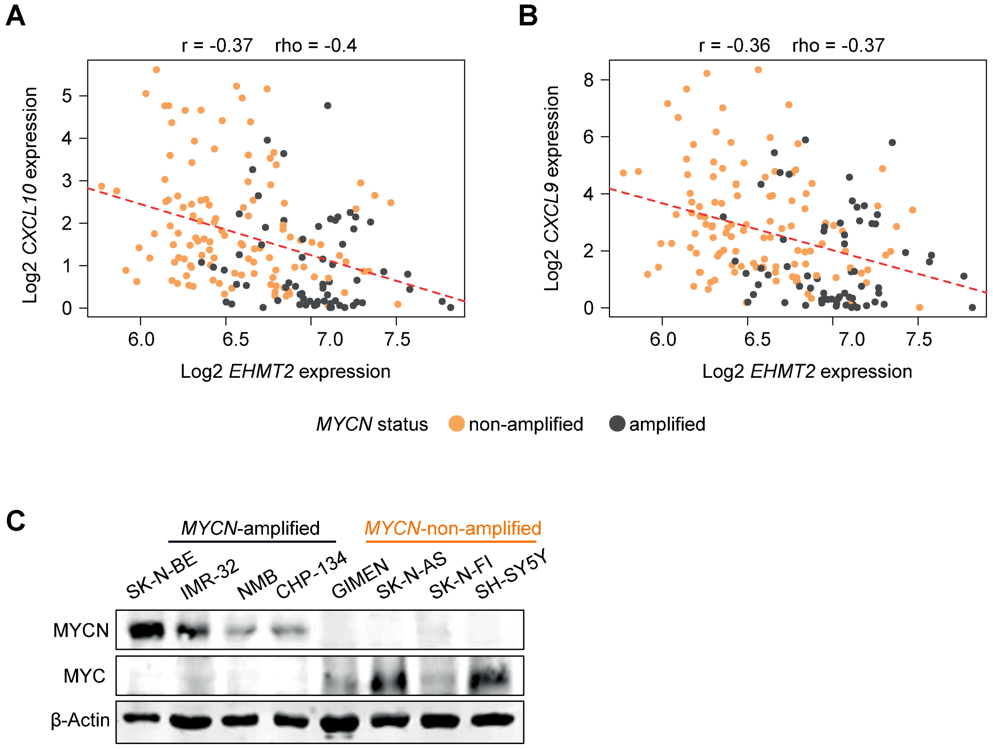

Online supplemental figure 1. Correlation of *EHMT2* with (A) *CXCL10* and (B) *CXCL9* expression in INSS 4 neuroblastomas (n = 181). r, Pearson's correlation coefficient; rho, Spearman's rank correlation coefficient. (C) Western blot for MYCN, MYC and β-actin of different NB cell lines. Representative blots of n=2.

Seier et al. online supplemental figure 2

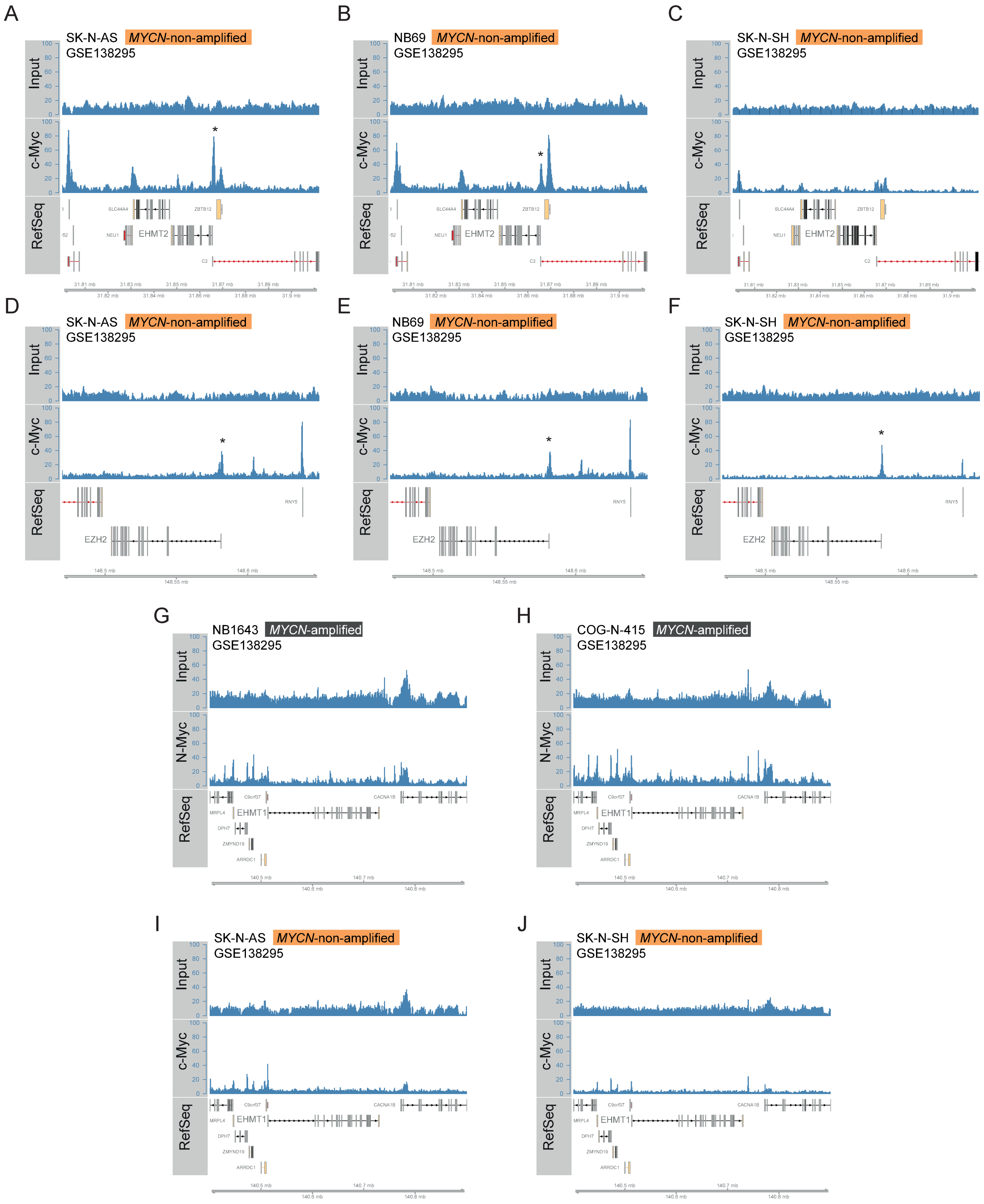

Online supplemental figure 2. ChIP-seq analysis of MYC and NMYC binding .in the genomic regions of *EHMT2*, *EZH2* and *EHMT1*. Plotted MYC (c-Myc) ChIP-Seq tracks obtained from GSE138295 showing the genomic region of EHMT2 in MYCN-non-amplified NB cells (A) SK-N-AS, (B) NB69 and (C) SK-N-SH. (D-F) as in A-C, but showing genomic region of *EZH2*. Plotted NMYC ChIP-Seq tracks obtained from GSE138295 showing the genomic region of *EHMT1* in *MYCN*-amplified NB cells (G) NB1643 and (H) COG-N-415. Plotted MYC (c-Myc) ChIP-Seq tracks obtained from GSE138295 showing the genomic region of *EHMT1* in *MYCN*-non-amplified NB cells (G) NB1643 and (H) COG-N-415.

Seier et al. online supplemental figure 3

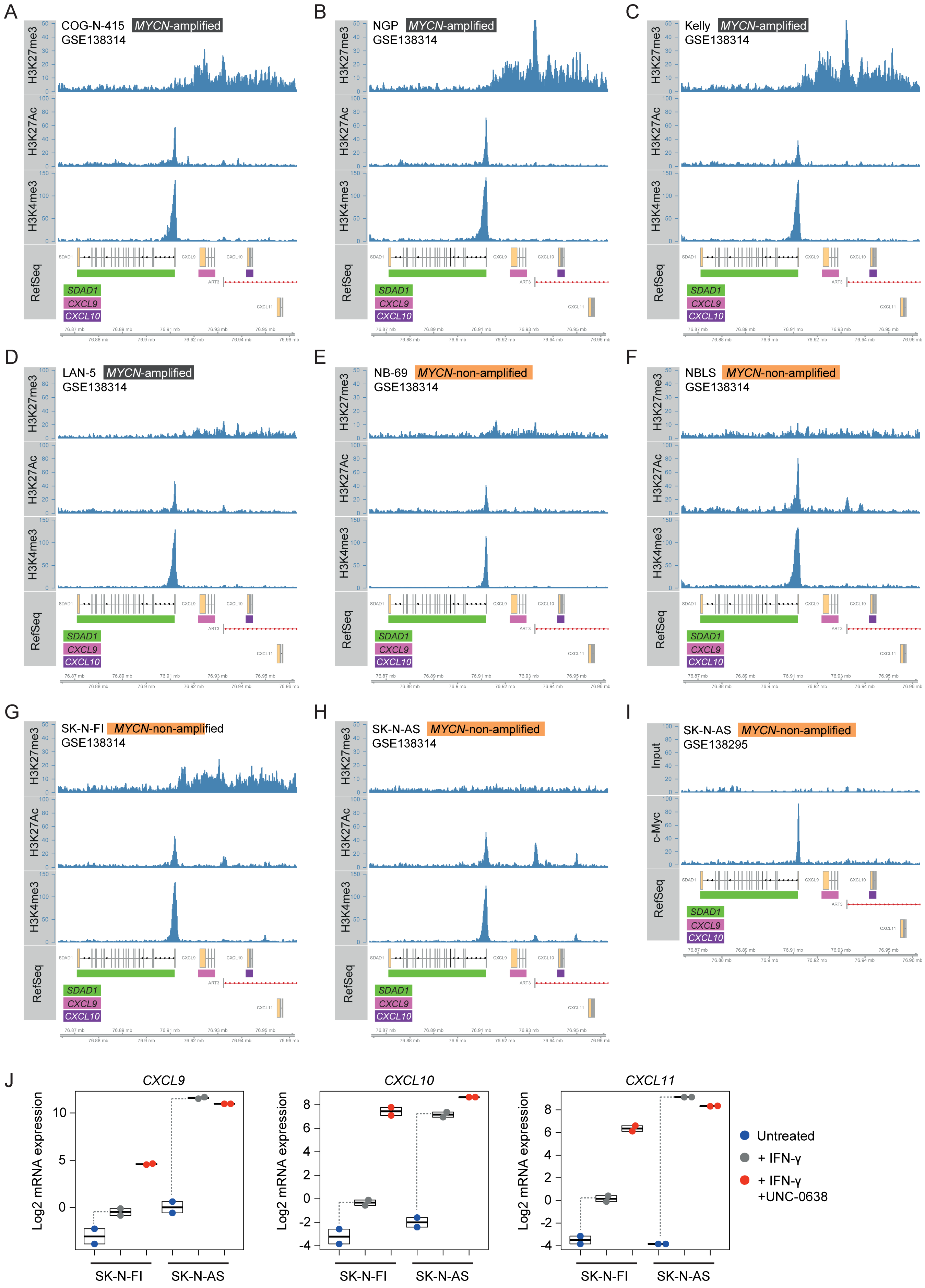

Online supplemental figure 3. H3K27me3, H3K27Ac and H3K4me3 histone ChIP-seq analysis of the genomic region of *SDAD1*, *CXCL9*, *CXCL10* and *CXCL11*. Plotted histone ChIP-Seq tracks obtained GSE138314 showing the genomic region of *SDAD1*, *CXCL9*, *CXCL10* and *CXCL11* in *MYCN*-amplified NB cells (A) COG-N-415, (B) NGP, (C) Kelly and (D) LAN-5 as well as in *MYCN*-non-amplified NB cells (E) NB69, (F) NBLS, (G) SK-N-FI and (H) SK-N-AS. (I) Plotted MYC (c-Myc) ChIP-Seq track and input obtained from GSE138314 showing the genomic region of *SDAD1*, *CXCL9*, *CXCL10* and *CXCL11* in SK-N-AS cells. (J) Log2 mRNA expression (3'mRNA-Seq) of *CXCL9*, *CXCL10* and *CXCL11* in *MYCN*-non-amplified NB cells SK-N-FI and SK-N-AS treated with IFN-γ, IFN-γ + UNC-0638 or left untreated. Dashed lines visualize chemokine gene induction by IFN-γ treatment alone. Data obtained from biological duplicates.

Seier et al. online supplemental figure 4

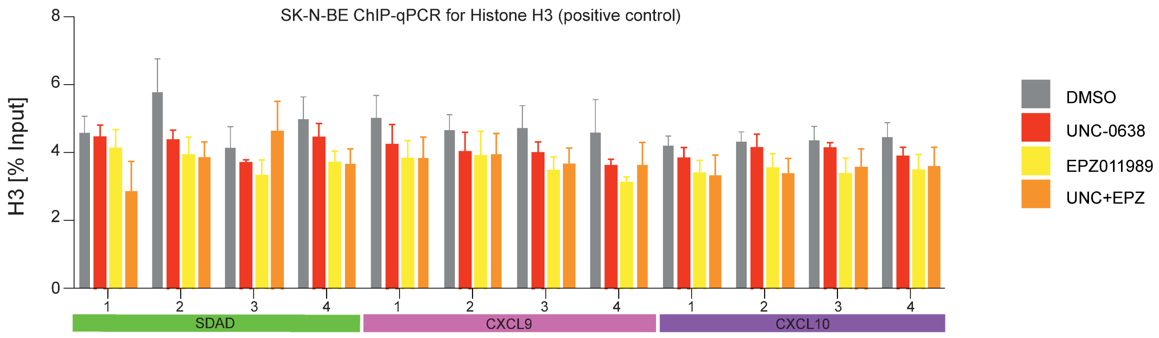

Online supplemental figure 4. Histone H3 control ChIP-qPCR quantified as % input.

Seier et al. online supplemental figure 5

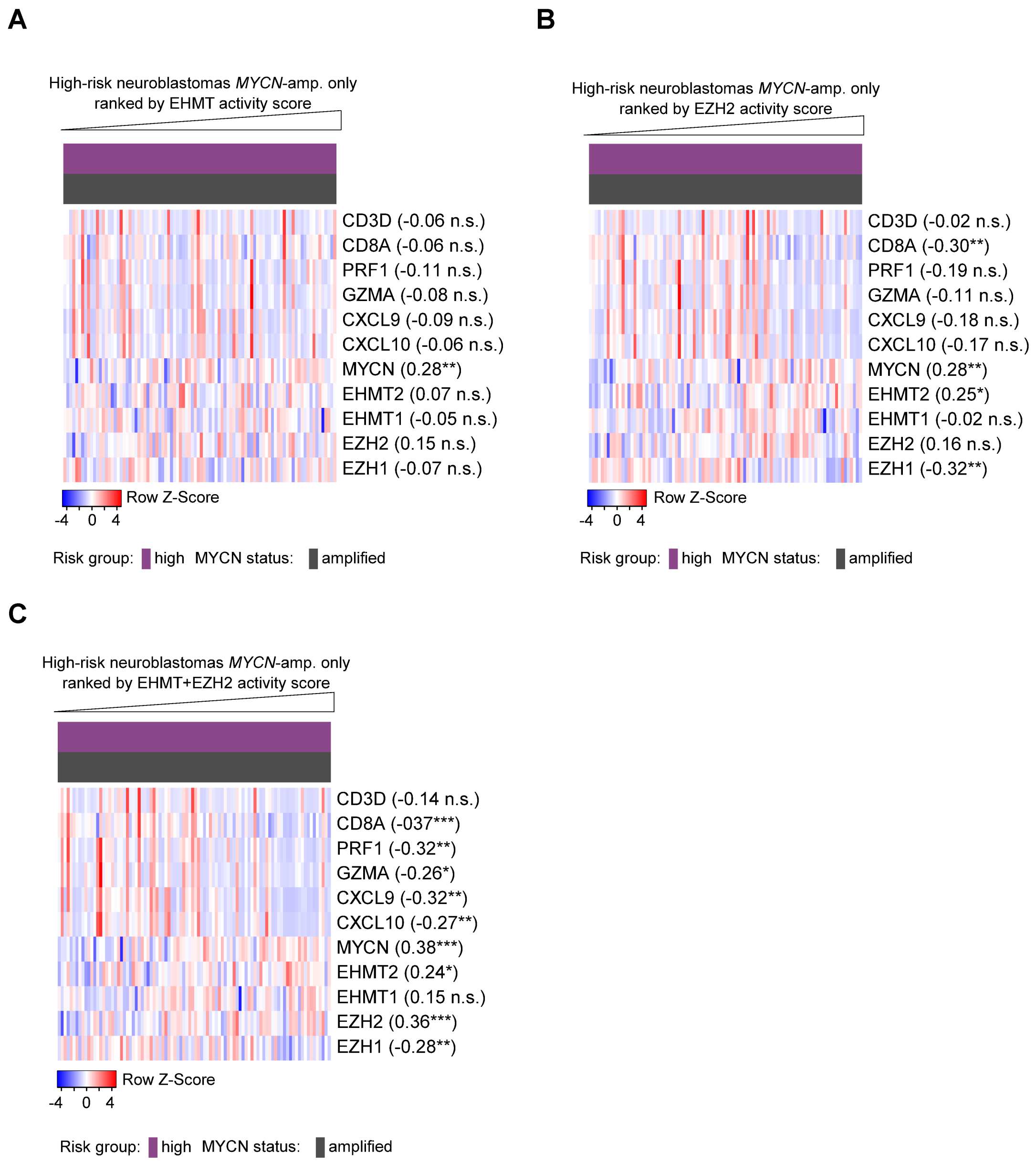

Online supplemental figure 5. EHMT and EZH2 acitivity scores in MYCN-amplified neuroblastomas.
(A-C) Heatmaps visualizing immune contexture marker genes (e.g. *CD8A*, *CXCL10*), *EHMT2/1*, *EZH2/1* and *MYCN* in high-risk NB samples (*MYCN*-amplified cases only) ranked by increasing activity scores of EHMT (A), EZH2 (B) and EHMT+EZH2 (C). Pearson correlation coefficients are indicated besides the names of the transcripts. Statistics: *, p<0.05; **, p<0.01; ***, p<0.001, n.s.; non-significan; two-sided t-test for Pearson's product moment correlation coefficient.
